# Engineered polyethylene terephthalate hydrolases: perspectives and limits

**DOI:** 10.1007/s00253-024-13222-2

**Published:** 2024-07-02

**Authors:** Fusako Kawai, Ryo Iizuka, Takeshi Kawabata

**Affiliations:** 1 Graduate School of Environmental and Life Sciences, Okayama University, 1-1-1 Tsushima-Naka, Kita-Ku, Okayama, 700-8530 Japan; 2 Graduate School of Science, The University of Tokyo, 7-3-1 Hongo, Bunkyo-Ku, Tokyo, 113-0033 Japan; 3 Graduate School of Information Sciences, Tohoku University, Aoba 6-3-09, Aoba-ku, Sendai, Miyagi 980-8579 Japan

**Keywords:** PET hydrolase, Engineering, Industrial biorecycling, Amorphous PET, Crystalline PET

## Abstract

**Abstract:**

Polyethylene terephthalate (PET) is a major component of plastic waste. Enzymatic PET hydrolysis is the most ecofriendly recycling technology. The biorecycling of PET waste requires the complete depolymerization of PET to terephthalate and ethylene glycol. The history of enzymatic PET depolymerization has revealed two critical issues for the industrial depolymerization of PET: industrially available PET hydrolases and pretreatment of PET waste to make it susceptible to full enzymatic hydrolysis. As none of the wild-type enzymes can satisfy the requirements for industrialization, various mutational improvements have been performed, through classical technology to state-of-the-art computational/machine-learning technology. Recent engineering studies on PET hydrolases have brought a new insight that flexibility of the substrate-binding groove may improve the efficiency of PET hydrolysis while maintaining sufficient thermostability, although the previous studies focused only on enzymatic thermostability above the glass transition temperature of PET. Industrial biorecycling of PET waste is scheduled to be implemented, using micronized amorphous PET. Next stage must be the development of PET hydrolases that can efficiently degrade crystalline parts of PET and expansion of target PET materials, not only bottles but also textiles, packages, and microplastics. This review discusses the current status of PET hydrolases, their potential applications, and their profespectal goals.

**Key points:**

• *PET hydrolases must be thermophilic, but their operation must be below 70 °C*

• *Classical and state-of-the-art engineering approaches are useful for PET hydrolases*

• *Enzyme activity on crystalline PET is most expected for future PET biorecycling*

**Graphical Abstract:**

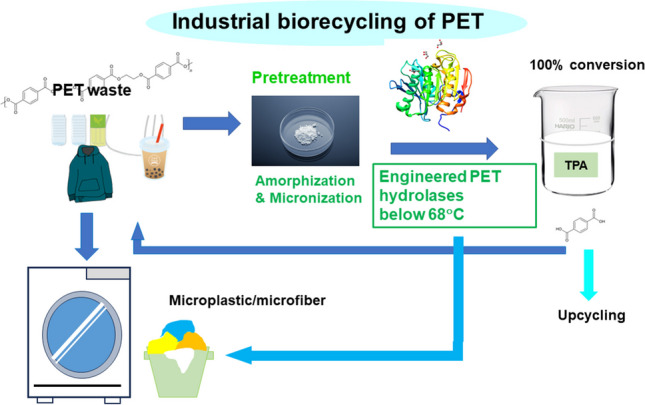

**Supplementary Information:**

The online version contains supplementary material available at 10.1007/s00253-024-13222-2.

## Introduction

Global plastic production began in the 1950s. Approximately 60% of plastics are discarded in the environment, where they persist, maintaining nearly the same properties and amounts as the original plastic due to their high durability and resistance (Geyer et al. 2017; Pandey et al. 2023). Plastic waste is ubiquitous and can be found from the heights of Mount Everest (Napper et al. 2020) to the depths of the Mariana Trench (Chiba et al. 2018). Polyolefins and polyesters make up most plastic waste (Geyer et al. 2017). Traditional methods of managing plastic waste, such as incineration and landfilling, are hindered by economic and environmental limitations. Incineration contributes to CO_2_ emissions, which exacerbates global warming. Landfills contribute to pollution in terrestrial and marine environments through the release of fragments and oligomers (Sang et al. 2020). These substances, when in the form of micro- or nanoparticles, are harmful to living organisms (Wang et al. 2020; Redondo-Hasselerharm et al. 2020) and can eventually enter the human food chain (Cverenkárová et al. 2021). Therefore, efficient management of plastic waste is becoming increasingly crucial as pollution concerns continue to rise.

Polyethylene terephthalate (PET) is a clear, strong, and lightweight plastic that is produced by polymerizing ethylene glycol and terephthalate. PET is the most produced polyester resin, with applications mainly in the manufacture of fibers for clothing and containers for food and beverages. It accounts for nearly 45% of single-use bottle production (Benyathiar et al. 2022; Wallace et al. 2020). PET bottles are recycled due to their high collection rates, which vary by country. However, PET bottles produced through mechanical recycling are of inferior quality compared to virgin bottles. Recycling is limited to a few cycles. In contrast, enzymatic depolymerization of PET can be practiced limitlessly, as it produces raw materials for PET synthesis, thus closing the loop from the synthesis of PET using raw materials to the depolymerization of waste PET to raw materials.

The initial aim of enzymatic PET degradation was to modify textiles, which required surface alteration without damaging the main body. However, unlike textile modification, complete degradation is required for PET waste. PET products have varying crystallinities: amorphous PET with a crystallinity of approximately 10% (used in packaging) and crystalline (semicrystalline) PET with a crystallinity of 30-40% (used in bottles and textiles). Amorphous PET is susceptible to enzymatic hydrolysis, whereas crystalline PET is resistant to such attacks. Currently, there are no practical PET hydrolases available for crystalline PET products. However, thermophilic PET hydrolases have almost reached practical levels for industrial PET biorecycling, resulting in nearly complete depolymerization of amorphous PET (Tournier et al. 2020; Arnal et al. 2023; Cui et al. 2024). The flexibility of the PET polymer depends on the temperatures. Below the glass transition temperature (*T*_g_), approximately 65-70 °C for PET in aqueous solution, the polymer is glassy. Above *T*_g_, the polymer becomes rubbery and flexible (Kawai et al. 2019), although the polymer undergoes recrystallization above 70 °C (Tournier et al. 2020). Enzymes cannot easily break down polymers in their glassy state. However, when the polymer chain becomes rubbery at temperatures above *T*_g_, but below 70 °C, it becomes susceptible to enzymatic attack. Enzymatic hydrolysis at ambient temperatures can detect depolymerized products, such as bis(hydroxyethyl)terephthalate (BHET), mono(hydroxyethyl) terephthalate (MHET), and terephthalate (TPA). PET includes the cyclic trimer of MHET, which is partially displayed on the surface along with any end/loop of a polymer chain and is exposed to surface modification through hydrolysis of ester bonds (Kawai et al. 2019). Enzymatic hydrolysis of PET at ambient temperature occurs only on the surface, resulting in minimal hydrolytic products. However, plastic pollution has attracted attention of the need to degrade the main block of PET waste. To achieve this, thermophilic PET hydrolases are used at temperatures above *T*_g_. These two types of enzymes are commonly employed as PET hydrolases. Some suggest to differentiate between two types of PET-hydrolyzing enzymes: PET surface-modifying enzymes and PET hydrolases (Kawai et al. 2019). This distinction has gained acceptance within the scientific community (Tournier et al. 2023).

This review presents the current situation of PET hydrolases for the biorecycling of waste PET. The feasibility of thermophilic and mesophilic enzymes as catalysts for biorecycling and their mutational improvements for future applications are discussed.

## PET hydrolases

PET, an aromatic polyester, can be hydrolyzed by the carboxylic ester hydrolase family (EC 3.1.1). A thermophilic aromatic polyesterase was first cloned from *Thermobifida fusca* (Müller et al. 2005), which belongs to the cutinase group in the carboxylic ester hydrolase family and can degrade the main body of PET at 55 °C. Cutinases catalyze the hydrolysis of cutin, a polyester found in plant cuticles. Some cutinases, such as the representative cutinase of *Fusarium solani pisi* (Nimchua et al. 2007; Ronqvist et al. 2009) can hydrolyze PET. Cutinases can readily hydrolyze the extreme surface area of PET at ambient temperature. However, enzymatic attack on the main body of PET is at a trace level, especially below *T*_g_ (~ 65−70 °C in water), as the polymer chain is unwavering below *T*_g_. Therefore, enzymes for biorecyling require thermostability (Kawai et al. 2019). Empirical data suggest that PET hydrolases require a melting temperature (*T*_m_) value at least 12 °C higher than their optimal temperature for catalytic longevity (Pfaff et al. 2022). Therefore, enzymes should have *T*_m_ values higher than 80 °C. Besides *T*_g_, the crystallinity of PET also influences its enzymatic degradation (Thomsen et al. 2022). Enzymatic degradation of crystalline PET (with a crystallinity of 30−40%) cannot be compared to that of amorphous PET (with a crystallinity of approximately 10%). Therefore, only detection of MHET, BHET, and TPA at low levels (< 1−2%) for amorphous PET is considered the surface modification level. For PET hydrolases, a large amount of the product must be released to destroy the main body of PET (1 mg of PET corresponds to ~ 5.2 μmol of MHET units). PET hydrolases, such as lipases, cutinases, esterases, carboxylesterases, and papain, can hydrolyze PET surfaces without damaging the main body. These enzymes are categorized as PET surface-modifying enzymes. PET hydrolases share common homologous structures, including α/β-hydrolases folds with a catalytic triad and a disulfide bond, regardless of their cutin-hydrolyzing activities. They are designated as cutinases or cutinase-like enzymes.

Although reports of PET hydrolase began in 2005 (Müller et al. 2005), they have not received worldwide attention, particularly from the public. In 2016, *Ideonella sakaiensis* was isolated, which was reidentified as *Piscinibacter sakaiensis* (Oren and Göker 2023). It can assimilate amorphous PET as a major carbon source at 30 °C and possesses a PET-hydrolyzing enzyme, *Is*PETase (Yoshida et al. 2016). The study compared the activity of *Is*PETase with that of other cutinases, such as TfCut2 (Müller et al. 2005) and LCC (Sulaiman et al. 2012) at 30 °C. The results showed that *Is*PETase was more effective than the other cutinases, indicating its potential as a hydrolase for decomposing waste PET, even at ambient temperatures. The possibility of enzymatic hydrolysis of PET has attracted the attention of scientists and the public. Mass communication has discussed this topic without detailed consideration. Many researchers have studied PET hydrolases, and some have noted that thermostability is a requirement for PET hydrolases. *Is*PETase has a low degradation ability because of its thermolability (Wei et al. 2019b). Several thermostable mutants of *Is*PETase have been discovered through mutational trials, including ThermoPETase (Son et al. 2019), DuraPETase (Cui et al. 2021), FAST-PETase (Lu et al. 2022a), HotPETase (Bell et al. 2022), and Z1-PETase (Lee et al. 2023).

Joo et al. (2018) classified bacterial PET hydrolases into two groups. Type I enzymes are thermophilic cutinase-type enzymes with one disulfide bond and the lack of an extensive β8-α6 loop. Type II enzymes, including *Is*PETase, are mesophilic and divided into IIa and IIb enzymes. The type IIa and IIb enzymes share two disulfide bonds and an extended β8-α6 loop but differ in the residues that constitute a putative secondary substrate-binding subsite (subsite II) and the extended loop. Based on this concept, PET hydrolases are classified according to their features (Table 1, Figs. S1 and S2). Type I enzymes share His164 and Phe243 residues in subsite II, except PHL7, where Phe is replaced with Leu (Sonnendecker et al. 2022). Type IIb enzymes have Trp and Ser/Thr at the corresponding positions, whereas type IIa enzymes have Trp and Phe/Tyr at the same position, except for PET2, where it is Trp and Trp (Meilleur et al. 2009; Danso et al. 2018; Nakamura et al. 2021), and *Ct*PL, where it is His and Phe (Chen et al. 2021; Li et al. 2023). His, Phe, Trp, and Tyr are aromatic amino acids and can replace each other. PET27 and PET30 from Bacteroidetes, recently characterized by Zhang et al. (2022a) have one disulfide bond. However, the position of one cysteine differs from that of typical type I enzymes. In addition, they include an extended loop and Trp and Ser residues in subsite II, like that of *Is*PETase. These enzymes are cold-active esterases, with PET27 having a C-terminal Por-sorting domain and PET 30 lacking a C-terminal Por-domain. Both enzymes exhibit high structural similarity to that of *Is*PETase, indicating that their core structures are type IIb enzymes. Recently, based on a sequence homology analysis of the NCBI protein database, a PET hydrolase candidate from an actinomycete, *Cryptosporangium aureantiacum* (*Ca*PETase: *T*_m_ value of 66.8 °C) was cloned, which has a type I-like structure except that His164 in LCC is replaced with Trp found in *Is*PETase, but Phe243 in LCC is conserved (subsite II) (Hong et al. 2023).

**Table 1:**
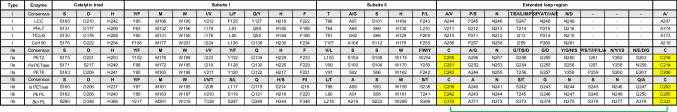
Comparison of active site residues of type I and type II enzymes

The GenBank accession numbers are as follows: LCC, HQ704839; PHL7/PES-H1, LT571446; TfCut2, AJ810119; Cut190, AB728484; PET2, ON416993; *Pa*PETase, OWL88088; PET6, FQUH01000018; *Is*PETase, WP_054022242; *Pb*PL, WP_047194864; *Bur*PL, OGB27210

Joo et al. (2018) reported that *Is*PETase outperforms type I enzymes at ambient temperatures due to two specific residues, Trp159 and Ser238, because the W159H/S238F mutation was found to reduce enzyme activity. Han et al. (2017) discovered that the substrate-binding residue Trp185 in *Is*PETase has multiple conformations (types A, B, and C), whereas the equivalent residue in type I enzymes only adopts a C-type conformation. This structural variation is due to the nearby residue Ser214, which is equivalent to His in type I enzymes. The smaller side chain of Ser214 allows Trp185 to move freely, whereas His restricts the rotation of the Trp side chain. *Is*PETase requires a flexible substrate-binding structure in subsite I to accommodate the inflexible PET molecule at ambient temperatures. However, above *T*_g_ temperature, the PET molecule becomes flexible, and type I enzymes require a rigid substrate-binding structure to capture it. Type IIb enzymes, in contrast, use an induced-fit mechanism to react, whereas type I enzymes use a lock-and-key mechanism. According to Austin et al. (2018), the W159H/S238F mutation in *Is*PETase improved PET degradation, contrary to the findings of Joo et al. (2018). *Is*PETase shares a high sequence identity with cutinases (> 43%) and adopts an α/β-hydrolase fold that harbors a catalytic triad and substrate-binding residues identical to those of canonical cutinases (Han et al. 2017; Joo et al. 2018). Unlike lipases, which have lids leading to interfacial activation, cutinase-like enzymes, including *Is*PETase, have open active sites without lids, in the same carboxyl ester hydrolase (EC 3.1.1).

Danso et al. (2018) predicted the global distribution of PET hydrolase homologs in bacterial and fungal phyla in marine and terrestrial environments, based on the Integrated Microbial Genome database. Biundo et al. (2017) reported that an esterase Cbotu_EstA cloned from *Clostridium botulinum* ATCC 3502, which has the structure of an α/β hydrolase with two lid domains, could marginally hydrolyze PET at 50 °C when the N-terminal region covering the catalytic site was truncated. PET46, a new enzyme with a lid that belongs to a feruloyl esterase, was recently cloned by a sequence-based metagenomic search from an uncultured deep-sea *Candidatus,* Bathyarchaeota archaeon. The enzyme exhibits a PET-degrading activity at 60 °C and a higher activity in BHET and MHET (Perez-Garcia et al. 2023). PET46 shares the core α/β-hydrolase fold with bacterial PET hydrolases but contains a unique lid domain common to feruloyl esterases (plant cell wall-degrading esterases). It is unclear whether this enzyme has any functions beyond the degradation of the plant cell wall in the deep-sea or if it is dormant. A feruloyl esterase homolog was cloned from *I. sakaiensis* as the enzyme to hydrolyze MHET (MHETase) that wild-type *Is*PETase produces from PET but cannot hydrolyze (Palm et al. 2019; Knott et al. 2020). However, MHETase is thermolabile as well as *Is*PETase. Polyesterases, Mors 1 and OaCut were cloned from the Antarctic bacteria *Moraxella* sp. strain TA144 and *Oleispira antarctic* RB-8, respectively, and hydrolyze PET at a rate comparable to *Is*PETase at 25 °C (Blázquez-Sánchez et al. 2022). The enzymes have Trp and Phe at the same position as His164 and Phe243 in subsite II of the LCC, which differs from Trp and Ser in *Is*PETase and have an extended loop length, like type II enzymes (Table 1). The proteins discussed in this passage belong to the type IIa family. However, unlike PET2 and *Ct*PL, which are also members of this family, their *T*_m_ values are like that of *Is*PETase. Mors 1 has a third disulfide bond near its N-terminal end, which may be an adaptation to low temperatures. Of note, OaCut lacks a cysteine in the corresponding position of the third disulfide bond.

Danso et al. (2018) was used as the basis for identifying a candidate PET hydrolase. Weigert et al. (2022) reported the prevalence PET6 homologs in Vibrios and characterized PET6 from *Vibrio gazogenes.* PET6 is like *Is*ETase, but its activity was lower than *Is*PETase. Notably, PET6 contains a third disulfide bond at its N-terminus. However, the role of the third disulfide bond requires further investigation. Type IIa enzymes are hybrids of type I and IIb enzymes. Type I enzymes can be thermophilic, whereas type IIb enzymes are mesophilic. Type IIa enzymes, such as PET2 and *Ct*PL, can be thermophilic, whereas Mors 1, OaCut, and *Pa*PETase (PE-H) are mesophilic. The behavior of PET hydrolases toward high, moderate, and low temperatures appears to be determined by small differences in their sequences, although they all share core structures as cutinases. PET hydrolysis requires (1) the catalytic triad and an oxyanion hole-forming aromatic residue, (2) an open active site, and (3) an appropriate size and amino acid composition of the catalytic site to bind the structural units of PET, such as MHET and BHET. PET hydrolysis, including surface-modification, may be easier than previously thought, as ester bonds can be hydrolyzed by various hydrolytic enzymes. Erickson et al. (2022) identified PET-active biocatalysts from natural diversity using bioinformatics and machine learning. They extracted 74 putative thermotolerant PET hydrolases (grouped into polyesterase-lipase-cutinases, cutinases, bacterial lipases, carboxylesterases, and peptidases), which revealed protein folds and accessory domains. Esterases, which are carboxylesterases without lids, are ubiquitous in organisms. Upon examination, many esterases have been observed to exhibit hydrolysis at various levels, as demonstrated by a metagenomic PET hydrolase from human saliva (Eiamthong et al. 2022). However, the hydrolytic activity for the main body of the PET structure is closely linked to the thermostability of PET hydrolases and their working temperature, which is close to the *T*_g_ value of PET, as described above.

**Fig. 1:**
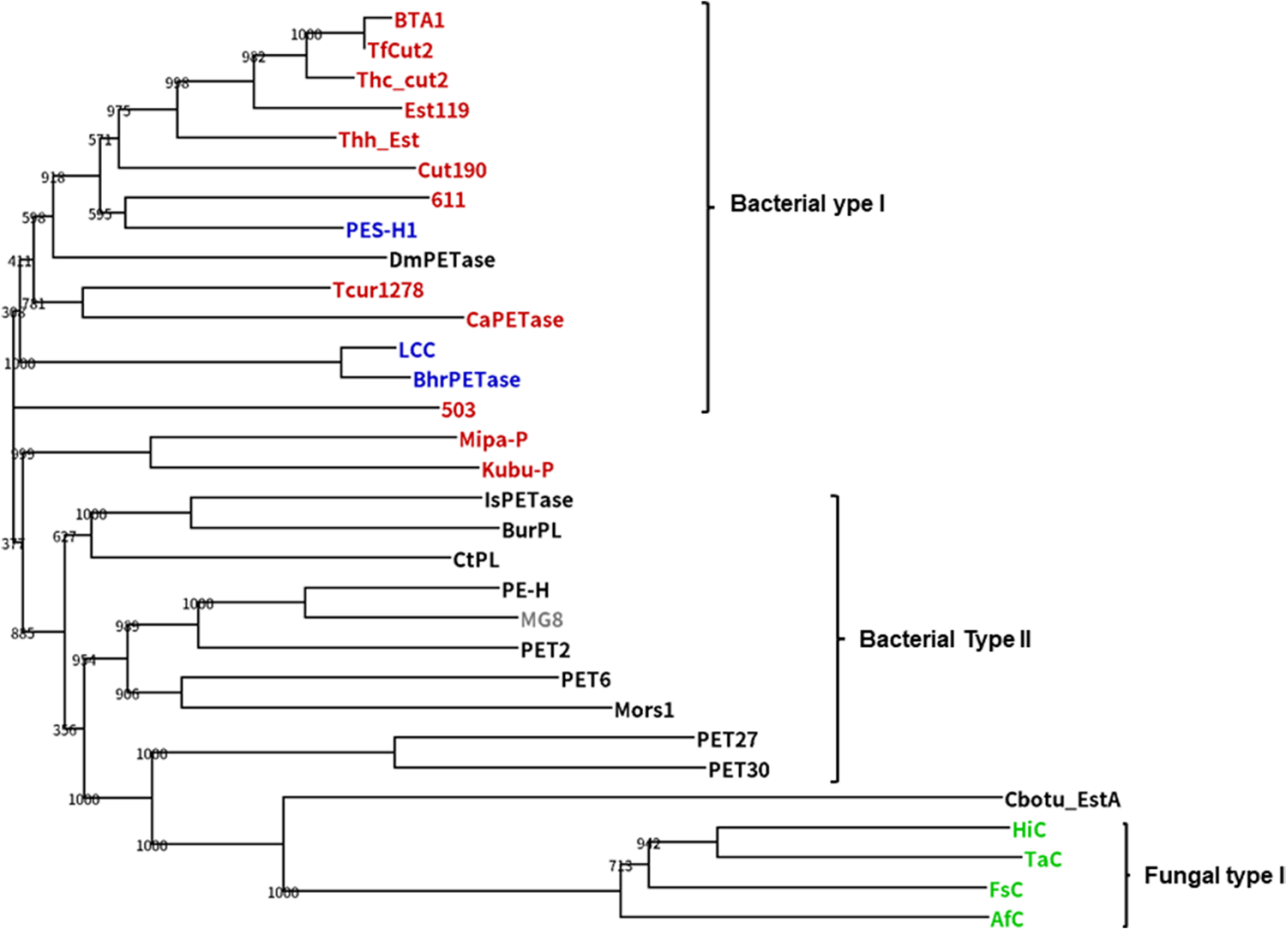
A phylogenetic tree of 31 PET hydrolases with activities. The GenBank accession numbers and PDB IDs of the hydrolases are summarized in Table S1. The groups “Fungi,” “Actinomycetes,” “Metagenome,” “Bacteria other than actionomycetes,” and “Others” are colored in green, red, blue, black, and grey, respectively. A multiple sequence alignment and the tree were calculated using the program ClustalW2 with default settings (Larkin et al. 2007) and visualized by the program Dendroscope 3.8.3 (Huson and Scornavacca 2012)

The development of high throughput measurements for PET-hydrolyzing activities (Weigert et al. 2021; Wei et al. 2022; Shi et al. 2023; Thomsen et al. 2023) made the screening of PET hydrolases far easier and quicker. Previous studies, starting from Müller et al. (2005), have accumulated information on the genetic sequences and the protein structures of PET hydrolases, which enabled the sequence-based screening and the computational/machine-learning search from metagenomic sources and databases such as the NCBI protein database and GenBank. Such a trial was first reported by Danso et al. (2018), proposing several PET hydrolase candidates, which were later characterized in detail, as described above (Nakamura et al. 2021; Weigert et al. 2022; Makryniotos et al. 2023). The number of PET hydrolases is rapidly increasing, based on state-of-the-art technologies (Erickson et al. 2022; Hong et al. 2023). Seo et al. (2024) extracted 2,064 non-redundant protein sequences grouped as polyesterase-lipase-cutinase in the database of the α/β hydrolase fold superfamily of proteins (Lenfant et al. 2013) and generated 1894 categories with 170 clusters, based on which 107 potential PETase were identified with sequence lineages. It is predicted that new enzymes capable of PET hydrolysis, possessing typical structural features of PET hydrolases (type I or II enzymes) or their mixed types, as well as additional unique features of the protein structure, will emerge. However, core structures, such as Cbotu_EstA, PET46, PET27, and PET30 will remain unchanged. PET hydrolases with confirmed PET hydrolytic activities included in this review are summarized as a phylogenetic tree in Fig. 1 and Table S1.

### Thermophilic PET hydrolases

Type I PET hydrolases are classified as cutinases. Research on cutinases focused on phytopathogenic fungi, such as *Botrytis cinereal* and *Fusrium solani pici* (Kawai et al. 2020). Later, attention shifted to polyesterases that work on synthetic polyesters, which are also part of the cutinase group. HiC, a commercialized cutinase from *Humicola insolens*, has optimal temperature of 75−80 °C and can efficiently hydrolyze amorphous PET at 70 °C (Ronqvist et al. 2009). Both enzymes belong to the cutinase/acetylxylan esterase family (InterPro: IPR000675) and make a distinct phylogenetic branch (Paysan-Lafosse et al. 2023). Brinch-Pedersen et al. (2024) selected 24 sequences from the InterPro IPR000675 family, homologous to HiC, among which the cutinase from *Thermocarpiscus australiensis* (TaC) performed better than HiC in the absence or presence of 300 mM NaCl. The salt dependence could be ascribed to electrostatic repulsion between TaC (negative surface) and semicrystalline PET powder (> 40% crystallinity). Several variants (less negative than wild-type) showed higher activity at 50 °C and 60 °C in salt-free buffer (pH 8). In addition, 15 fungal cutinases were cloned by a sequence homology analysis from the NCBI database (Lee et al. 2024). A phylogenetic tree analysis with two known fungal cutinases (HiC and FsC) showed that these fungal cutinases are divided into two distinct types. Type I comprises HiC, FsC, and 10 cutinases with notable sequence similarities, encompassing all reported PET-degradable cutinases. Type II showed lower sequence homology with type I and significant divergence within them. This could be a standard for fungal cutinases. The InterPro IPR000675 family belongs to the α/β hydrolases, but the size of fungal cutinases is smaller than that of bacterial cutinases, comprising only 5 β-strands, which are covered by 5 α-helices. Bacterial cutinases comprise 9 β-strands, which are covered by 8 α-helices. A cutinase from *Aspergillus fumigatiaffinms* (AfC) had a potent PET-hydrolyzing activity, and its variant (AfC^6P^) completely decomposed the post-consumer PET film within 12 days at 60 °C, but its availability to biorecycling remains to be studied. These reports expanded the diversity of fungal PET hydrolases, but information on fungal PET hydrolases is limited compared to those on bacterial ones. It must need further work whether the fungal cutinases can be equivalent to bacterial ones (Arnal et al. 2023) or outperform them or not.

According to Danso et al. (2018), Actinobacteria are the main hosts of PET hydrolase genes in terrestrial environments. Thermophilic PET hydrolases have been obtained from various Actinomyces, including the genera *Thermobifida*, *T. fusca*, *T. alba*, *T. cellulosilytica*, and *Saccharomonospora viridis* (Kawai et al. 2020; Kawai 2021) (Table S2). The thermophilic enzyme LCC was cloned from plant compost using a metagenomic approach (Sulaiman et al. 2012). Subsequently, BhrPETase was cloned from the thermophilic bacterial phylum *Chloroflexi* derived from a subsurface geothermal stream using a metagenomic approach. The enzyme has a 94% sequence identity with LCC and exhibits a slightly better PET-hydrolyzing activity than LCC (Xi et al. 2021). *Chloroflexi* has been identified as one of the dominant members in compost microbial communities (Lu et al. 2022b). Given that the sequence identity of LCC with Actinomycete cutinases is less than 60%, LCC is likely mainly derived from *Chloroflexi* rather than Actinomycetes. Furthermore, PHL7 was recently cloned from plant compost using a metagenomic approach (Sonnendecker et al. 2022). Recently, a thermophilic polyesterase *Dm*PETase was cloned from *Deinococcus maricopensis.* Its presence was suggested by Danso et al. (2018). *Dm*PETase shares 60% identity with hydrolases of *Thermobifida* species, and LCC, and contains one disulfide bond. Makryniotos et al. (2023) classified this enzyme as a type I enzyme. Seo et al. (2024) navigated a natural landscape of ester-based plastic hydrolases from a library including 25,418 sequences using a sequence homology network and clustered 1894 PET hydrolase candidates into 170 lineages. Based on this result, they discovered novel PET hydrolases (Mipa-P and Kubu-P) with high catalytic ability and high thermal stability originated from actinomycetes, *Micromonospora pattaloongensis* DSM 45254 and *Kutzneria buriramensis* IDSM 45791, from which no PET hydrolases have been documented (Fig. S3). Both enzymes have one disulfide bond and no extended loop. They share most of the local sub-network structures of *Ca*PETase. However, His 164 and Phe243 in LCC, characteristic of type I enzymes, are not conserved. PET2 (Nakamura et al. 2021) and *Ct*PL from *Caldimonas taiwanensis* (Chen et al. 2021; Li et al. 2023) are thermophilic type IIa enzymes (Table 1).

The history of 20 years’ research on PET hydrolases accumulated uncountable sequences from bacteria and fungi and the number is annually increasing. Computer-aided and machine-learning screening from databases would bring novel enzymes from unexplored sources, probably resulting in expansion of a variety of PET hydrolases, as described above.

### Mesophilic PET hydrolases

Joo et al. (2018) categorized type IIb enzymes based on the findings of *Is*PETase. Danso et al. (2018) predicted the presence of various IIb enzymes. The ability of microorganisms to grow on PET at ambient temperatures has been demonstrated in previous studies (da Costa et al. 2020; Moyses et al. 2021; Qiao et al. 2022). Furthermore, various sources have produced type IIb enzyme clones with thermostability comparable to that of *Is*PETase (Bollinger et al. 2020; Chen et al. 2021; Eiamthong et al. 2022) (Table S3). Table 1 describes the characteristics of type IIb enzymes, which include an additional disulfide bond, a longer extended loop than type I enzymes, and Trp-Ser/Thr at the corresponding positions of His164 and Phe243 in LCC. Both type I and II enzymes have a Tyr/Phe-Met-Trp substrate-binding motif in subsite I, which acts as an aromatic clamp (Danso et al. 2018). Two cold-active esterases PET27 and PET30, were cloned from Bacteroides *Aequorivita* sp. and *Kaistella jeonii*, respectively (Zhang et al. 2022a). Both enzymes lack additional disulfide bonds, like type I enzymes, but have a longer extended loop than type I and contain Trp-Ser at the corresponding positions of His164 and Phe243 in the LCC, which are characteristic features of type IIb enzymes. PET30 has a Phe-Met-Tyr motif at subsite I, which differs from the consensus sequences of Tyr/Phe-Met-Trp, whereas PET27 has a Tyr-Met-Trp motif. Tyr can replace Trp as an aromatic clamp. Furthermore, some type IIa enzymes, such as *Pa*PETase/PE-H (Bollinger et al. 2020) and MG8 (Eiamthong et al. 2022) are mesophilic, whereas type IIa PET2 (Nakamura et al. 2021) and *Ct*PL from *C. taiwanensis* (Chen et al. 2021; Li et al. 2023) are thermophilic. As explained above, type IIa enzymes are hybrids of type I (thermophilic) and IIb (mesophilic) enzymes, characterized by their amino acid sequences and resistance to temperature.

A large amount of information has been accumulated on PET hydrolases, specifically regarding their sequences and 3D structures. Various PET hydrolases have been discovered, differing slightly from typical type I and II enzymes. Some may have additional structures like a lid or a Por-domain. However, these enzymes conserve the core structures as described above. It is widely agreed that PET hydrolases must have sufficient thermostability for their use in the industrial biorecycling of PET (Arnal et al. 2023). Additional structures, such as the extended loop and lid, may pose a challenge and a simple core structure may be adequate for PET hydrolysis. However, wild-type I enzymes are not adequate for PET biorecycling. Mutational improvements have been attempted to enhance the thermostability and activity of enzymes. Recent advancements in creating PET hydrolase variants are based on various technologies such as classical rational engineering/directed evolution and state-of-the-art computational/machine-aided engineering. This has led to a rapid increase in publications for the variants.

## Engineering of type I PET hydrolases

Enzymes are crucial for industrial-scale enzymatic PET depolymerization, with PET hydrolysis enzymes being the most important. Research has focused on the engineering of these enzymes to improve their efficiency and optimize the expression system of PET hydrolase genes and reaction conditions. The efficiency of the enzyme is influenced by both the crystallinity and the size of the PET substrate. Arnal et al. (2023) summarized critical parameters for enzymatic depolymerization in industrial settings. This section focuses on improving enzyme efficiency through mutation.

### Metal-binding sites

These concentrations are much higher than the typical concentrations of metal ions required for prosthetic groups. The activity and thermostability of several PET hydrolases are increased by the presence of these ions. Thumarat et al. (2012) observed that Est119 from *Thermobifida alba* AHK119 showed increased activity in the presence of high concentrations of Ca^2+^, Mg^2+^, and Mn^2+^. The activity and thermostability of Cut190 (Kawai et al. 2014), LCC (Sulaiman et al. 2014), TfCut2, and TfCut1 (Then et al. 2015) are increased by the presence of Ca^2+^ and Mg^2+^. These enzymes have negatively charged amino acid residues that bind cations at the same positions: Asp174, Asp204, and Glu253 in TfCut2 (Then et al. 2015) and ThCut1 (Zhang et al. 2022b), but Asp174 is replaced with Glu in LCC and Cut190. The introduction of a disulfide bond into the metal-binding site enhances the thermostability and activity of TfCut2 (Then et al. 2016), Cut190 (Oda et al. 2018), LCC (Tournier et al. 2020), and ThcCut1 (Zhang et al. 2022b). The substitution of Ca^2+^ with a disulfide bond enables the use of a phosphate buffer (precipitated with Ca^2+^ or Mg^2+^) for the enzyme reaction, which is more cost-effective for industrial applications. Cut190 is the only Ca^2+^-activated PET hydrolase. It has inactive and active forms in the absence and presence of Ca^2+^, respectively (Miyakawa et al. 2015). This is due to the absence of an amino acid in the β1-β2 loop of Cut190, compared to other enzymes (Oda et al. 2018) (Fig. S2). Cut190 has three Ca^2+^-binding sites: site 2 is a common cation-binding site for cutinases, whereas site 1 is required for its unique activation by Ca^2+^ (Numoto et al. 2018). The crystal structure of *Is*PETase^W159F^ (PDB ID: 6ILX) is like that of Cut190 (PDB ID: 5NZO), particularly in the Ca^2+^-binding sites (Liu et al. 2019; Liu et al. 2023). Two metal-binding sites have been identified on the surface of PHL7, which differs from sites 2 and 3 of Cut190 and may serve as future mutagenesis targets to increase its thermostability, as well as other PET hydrolases (Richter et al. 2023).

### Substrate-binding sites

Joo et al. (2018) conducted a molecular docking study of *Is*PETase with 2-hydroxyethyl-(monohydroxyethyl terephthalate)_4_ (2-HE(MHET)_4_) and identified four substrate-binding subsites: subsites I, IIa, IIb, and IIc. Kawabata et al. (2017) also proposed that amino acids are responsible for binding the PET model substrate to Cut190. According to their model, the substrate is expected to be in an extended form, which differs from the bent form of 2-HE(MHET)_4_ that interacts with *Is*PETase. The amino acids involved in substrate-binding are in subsite I and part of subsite II according to this model. The substrate-binding cleft can accommodate one or two MHET units. Wei et al. (2019b) stated that whereas the catalytic triad of *Is*PETase could correctly access the ester bond in 2-HE(MHET)_4_ located between subsites I and IIa, subsites IIb and IIc were unlikely to interact with the other two MHET units, as suggested by Joo et al. (2018). Furthermore, Richter et al. (2023) hypothesized that the weak interaction between the aromatic phenylene moieties in PET and the surrounding hydrophobic amino acid residues facilitates substrate binding required for subsequent enzymatic hydrolysis of PET, rather than the perfect accommodation of a specific conformation of a polymer segment. They compared the amino acids in subsites I and II of type I and II enzymes. The authors suggest that subsite I is the primary contributor to binding, interacting with one PET moiety to achieve a productive conformation at the catalytic serine. In contrast, subsite II mainly contributes to the initial substrate binding and guides the PET chain toward the active site. Thus, subsite II may act as a guiding channel with loose interactions, transferring the new substrate chain produced by ester hydrolysis to subsite I. However, more research is necessary to confirm this hypothesis.

#### Subsite I

**Fig. 2:**
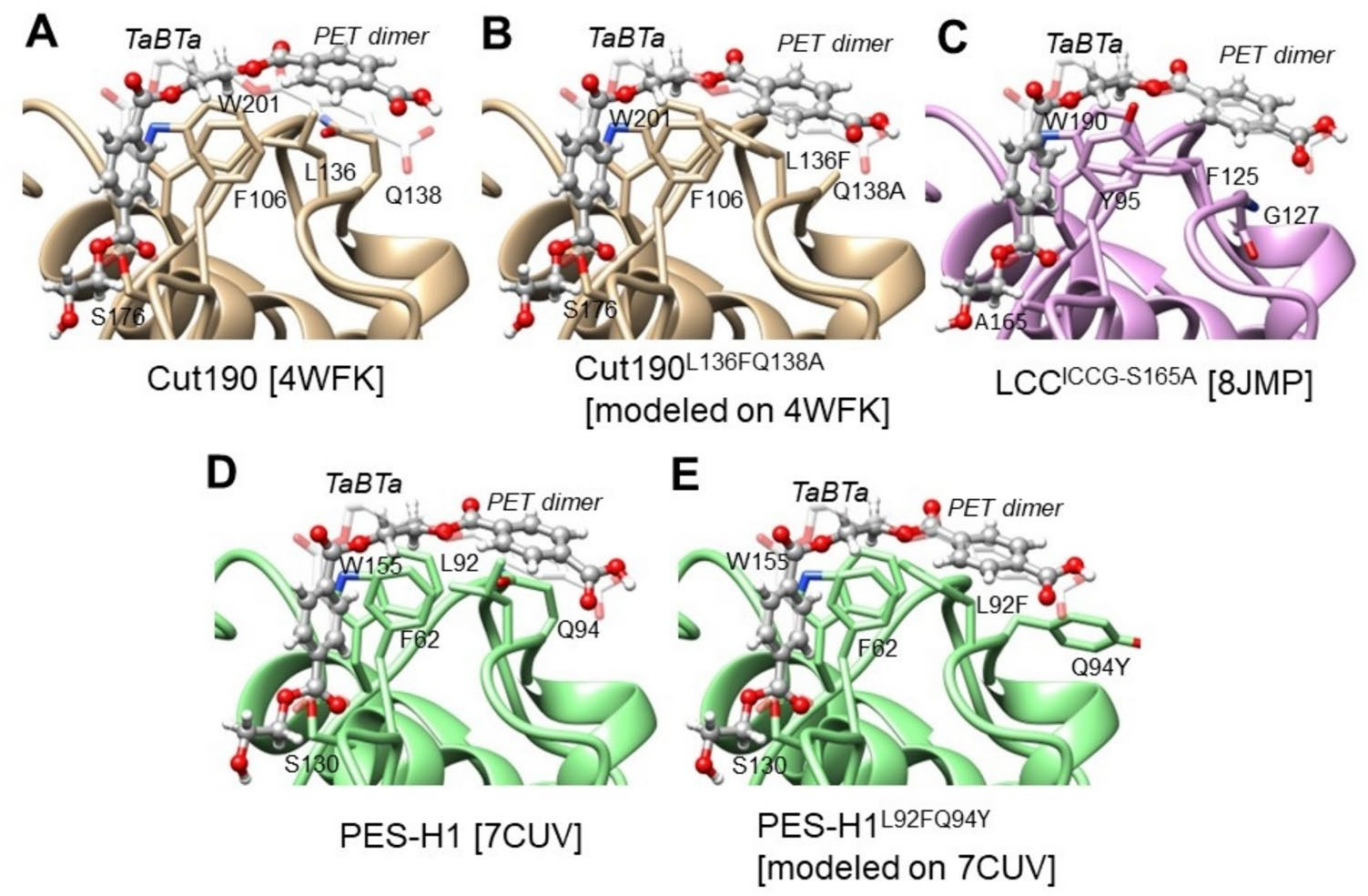
3D structure models of PET hydrolases with a PET dimer. PET dimer-bound models were built based on the structure of 1,4-butnediol diterephthalate (TaBTa) in LCC^ICCG-S165A^ (PDB ID: 8JMP) as a template, as described in Supplementary Information (Scheme S1 and Fig. S4). The superimposed TaBTa template molecules are indicated in white transparent stick models. The PET dimer models, shown in gray ball-and-stick models, were generated by fitting on the template and energy minimization. **A** Cut190 (PDB ID: 4WFK). **B** Cut190^L136FQ138A^. The mutated structure was built on PDB ID: 4WFK. **C** LCC^ICCG-S165A^ (PDB ID: 8JMP). **D** PES-H1 (PDB ID: 7CUV). **E** PES-H1^L92FQ94Y^. The mutated structure was built on PDB ID: 7CUV

The amino acid compositions at subsite I were similar for type I enzymes (Table 1). The residues His218 and Phe222 in the LCC were highly conserved. In PHL7 and Cut190, Tyr95 of LCC was replaced with Phe; in PHL7, TfCut2 and Cut190, Val212 of LCC was replaced with Ile; Phe125 of LCC was replaced with Leu in other cutinases; and in BhrPETase, Tyr127 in LCC was replaced with Phe, whereas other cutinases have Gln at this position. In Cut190, replacing Gln138 with a small amino acid, Ala, improves activity by avoiding the atomic clashes of substrates with the residue positioned toward the extension of PET as a barrier (Fig. 2A and B) (Oda et al. 2018). Similarly, replacing Tyr127 with Gly in LCC improves the activities of LCC^ICCG^ and LCC^WCCG^, as it is located at the same position as Gln138 in Cut190 (Tournier et al. 2020) (Fig. 2C).

TfCut2, Cut190, BhrPETase, and PHL7 all have Leu at position Phe125 in the LCC (Table 2). According to Kawai et al. (2022), the activity of Cut190 increases when Leu136 is mutated into Phe. This position is close to Gln138 (Cut190) and appears to be the exit of the polymer chain. Therefore, aromatic amino acids such as Phe may interact more with aromatic terephthalic acids than with hydrophobic amino acids, such as Leu. Pfaff et al. (2022) modified Leu92 and Gln94 of PHL7 (now called PES-H1) to Phe and Tyr, respectively, at the corresponding positions of Leu136 and Gln138 in Cut190. This modification improved hydrolytic activity against amorphous PET films and pretreated PET waste, possibly due to the creation of an aromatic channel with a known aromatic clamp (Trp155 and Phe62) (Fig. 2D and E). It is suggested that Ala/Gly may be a better substitution for Gln94, as it removes a barrier, and that Phe may be better for intensifying the aromatic channel. Table 2 shows that His218 and Phe222 in LCC are highly conserved among thermophilic PET hydrolases. These two residues are characteristic of type I enzymes. However, in BhrPETase, these two amino acids are mutated into Ser and Ile (TurboPETase; BhrPETase^H218S/F222I/A209R/D238K/A251C/A281C/W104L/F243T^) (Cui et al. 2024). Ser and Ile are the same residues as those in *Is*PETase and appear to weaken the interaction with the aromatic polymer, in contrast to those of His and Phe.

**Table 2:** Engineering of subsites I and II residues in type I enzymes

Type	Enzyme	Subsite I								Subsite II					Extended loop region									
I	PHL7/PES-H1	F6313S	M132	W156	I179	L93	Q95	H185	F189	T64	A65	S69	H130	L210	V211	S212	N213	T214	P215	D216	-	-	-	A174
	PES-H1mut	F6313S	M132	W156	I179	*F93*	*Y95*	H185	F189	T64	A65	S69	H130	L210	V211	S212	N213	T214	P215	D216	-	-	-	A174
I	LCC	Y95	M166	W190	V212	F125	Y127	H218	F222	T96	A97	S101	H164	F243	A244	P245	N246	S247	N248	N249	-	-	-	A207
	LCC^ICCG^	Y95	M166	W190	V212	F125	*G127*	H218	F222	T96	A97	S101	H164	*I243*	A244	P245	N246	S247	N248	N249	-	-	-	A207
	LCC-A2	Y95	M166	W190	V212	F125	*G127*	*Y218*	F222	T96	A97	S101	H164	*I243*	A244	P245	N246	S247	*D248*	N249	-	-	-	A207
I	BhrPETase	Y95	M166	W190	V212	L125	F127	H218	F222	T96	A97	S101	H164	F243	A244	P245	N246	S247	P248	N249	-	-	-	A207
	TurboPETase	Y95	M166	W190	V212	L125	F127	*S218*	*I222*	T96	A97	S101	H164	*T243*	A244	P245	N246	S247	P248	N249	-	-	-	A207
I	TfCut2	Y60	M131	W155	I178	L90	Q92	H184	F188	T61	G62	S66	H129	F209	A210	P211	N212	I213	P214	N215	-	-	-	A173
	TfCut2mut	Y60	M131	W155	I178	L90	Q92	H184	F188	T61	*A62*	S66	H129	*I209*	A210	P211	N212	I213	P214	N215	-	-	-	A173
I	Cut190	F106	M177	W201	I224	L136	Q138	H230	F234	T107	A108	S112	H175	F255	A256	P257	N258	I259	P260	N261	-	-	-	A219
	Cut190mut	F106	M177	W201	I224	*F136*	*A138*	H230	F234	T107	A108	S112	H175	F255	A256	P257	N258	I259	P260	N261	-	-	-	A219
IIb	*Is*PETase	Y87	M161	W185	I208	L117	Q119	S214	I218	T88	A89	S93	W159	S238	C239	A240	N241	S242	G243	N244	S245	N246	Q247	C203

The residues of wild-type type I enzymes and their variants were compared at subsites I and II. Mutated residues are shown in italics

PES-H1mut: PES-H1^L92F/Q94Y^

LCC^ICCG^: LCC^Y127G/D238C/F243I/S283C^

LCC-A2: LCC^Y127G/H218Y/D238C/F243I/N248D/S283C^

TurboPETase: BhrPETase^H218S/F222I/A209R/D238K/A251C/A281C/W104L/F243T^

TfCut2mut: TfCut2^G62A/F209I/F249R^

Cut190mut: Cut190^L136F/Q138A/S226P/R228S/D250C/E296C/Q123H/N202H/K305del/L306del/N307del^

#### Subsite II

Subsite II contains two residues, His164 and Phe243, which are characteristic of type I PET hydrolases. A single mutation of F243I decreased the *T*_m_ value of LCC, but LCC^ICCG^ had its *T*_m_ value increased by 9.3 °C (94.0 °C). The template for TurboPETase (BhrPETase^H218S/F222I/A209R/D238K/A251C/A281C/W104L/F243T^) is BhrPETase, which has a 94% identity with LCC. However, it has Thr243 instead of Phe243, which is the same as type IIb *Pb*PL (Table 1). A disulfide bond was introduced in Ala251 and Ala281, which differs from Asp238 and Ser283, respectively, in LCC^ICCG^. However, both disulfide bonds likely played similar roles in TurboPETase and LCC^ICCG^. TurboPETase could break down 200 g/L of pretreated post-consumer PET bottles at 65 °C for 8 h. The improved performance of TurboPETase can be attributed to its PET-binding groove (Ser218/ILe222/Thr243/Leu104), which is more flexible and allows for more precise targeting of attacks (Cui et al. 2024). The *T*_m_ value of BhrPETase was 101 °C, which is higher than LCC. TurboPETse, in contrast, had a *T*_m_ value of 84 °C, which is like that of LCC. This is because of the replacement of Phe243 with Thr243 (subsite II) and a pair of His218 and Phe222 with Ser and Ile (subsite I) (Table 2). Higher *T*_m_ values result from the greater rigidity of proteins. However, maintaining sufficient thermostability while allowing for some flexibility may lead to better performance in the hydrolysis of PET. Replacement of Phe243 with Ile or Trp in LCC resulted in better performance (LCC^ICCG^ and LCC^WCCG^ with *T*_m_ values of 94.0 °C and 98.0 °C, respectively) than in LCC. The same positive effect of replacing Phe with Ile was observed in ThcCut1^AICCG^ (Zhang et al. 2022b). Although the core structure of PET hydrolases is similar in both type I and II enzymes, there are differences in their sequences, except for important motifs. These differences may be related to each other and result in recombinant expression of genes, integrity of protein structure, thermostability, and activity. PHL7 (PES-H1) is a type I PET hydrolase, but it contains Leu (Leu210) at the position of Phe243 in the LCC. The substitution of Leu with Thr (L210T) resulted in significantly higher activity, whereas substitution with Phe (L210F) decreased activity by approximately half (Richter et al. 2023). A similar effect was observed on TurboPETase upon replacement with Thr, which enabled the enzyme structure to be more flexible. The mutant maintains sufficient thermostability for PET hydrolysis, although with a slightly lower *T*_m_ value of approximately 1 °C. The substitution of Ile/Leu for Phe and Thr for Ile/Leu at this position may be more effective in providing flexibility to subsite II by loosening its interaction with PET. Although these type I enzymes share core structures, there are subtle differences among them. The findings from LCC, PHL7 (PES-H1) and TurboPETase suggest that any variants in subsites I and II could cause improved performance, unless the core structure of the proteins is destroyed, and the interaction of amino acids with PET is well tuned. Pirillo et al. (2023) created LCC^S101N/F243T^ with *T*_m_ value of 76.6 °C (lower by 4.2 °C than LCC), which showed better activity at 55 °C for untreated post-consumer PET waste than LCC. The basic reaction mechanism of thermophilic enzymes must be a lock and key mechanism as described above, but subtle tuning of the substrate-binding groove performs better.

Table 2 shows that TfCut2 contains Gly62, whereas other type I and type IIa enzymes have Ala at the same position. The activity of TfCut2 is inhibited by the hydrolytic products BHET and MHET, but this inhibition is overcome by the G102A (G62A without a signal peptide) mutation (Wei et al. 2016). Furukawa et al. (2019) also found that the G62A mutation, together with the Phe209 mutation (corresponding to Phe243 in LCC), increased the activity of TfCut2. TfCut2^F209A^, a single mutation, showed improved activity. Mrigwani et al. (2023) created a G62A/F209I/E249R mutant of TfCut2. Therefore, it is possible to replace Phe with Ile/Leu, Thr and Ala at this position. However, the effect of Ala has not been compared to that of Ile/Leu and Thr. According to Chen et al. (2022), a combination of H184S/Q92G (subsite I) and F209I/I213K (subsite II) mutations in *T. fusca* cutinase (TfCut^D204C/E253C^) increased enzyme activity by approximately 30-fold. This combined mutation is useful for LCC, Est119 and BhrPETase. H184S corresponds to S214 of *Is*PETase. LCC^ICCG^ contains Q92G and F209I mutations. However, the *T*_m_ values of these mutants have not yet been determined, making it difficult to evaluate them using other variants of TfCut2, LCC, Est119 and BhrPETase. Furthermore, we could not locate a reference for the original template enzyme (Zhu et al. 2021) on the Internet, so we could not confirm the details of the strains and template enzymes.

LCC^ICCG^ was further mutated by docking-assisted engineering (Zheng et al. 2024). The resultant best variant of LCC^ICCG^ (LCC-A2: LCC^ICCG/H218Y/N248D^: *T*_m_ value of 95.25 °C, higher by 1.11 °C than LCC^ICCG^) has the widened substrate channel between subsite I (H218Y) and subsite II and showed the improved ligand interactions near the active center (N248D), by which the variant depolymerized > 90% of the pretreated, post-consumer PET waste (200 g/kg) within 3.3 h at 78 °C. Under the rapid depolymerization process, recrystallization might not affect significantly, as the performance of the variant was better at 78 °C than at 72 °C.

### Other sites

Besides the direct substrate-binding sites, the mutation of the surface charge was tried for *Ca*PETase having Trp (characteristic of type II enzymes) at the position of His164 in LCC (characteristic of type I enzymes). Hong et al. (2023) introduced disulfide and hydrogen bonds and modified the protein surface charge to create a rationally designed mutant (*Ca*PETase ^M9^). *Ca*PETase ^M9^ has a *T*_m_ value of 83.2 °C and displayed more than 90% depolymerization of post-consumer PET powder (3.75 g/150 mL) at 55 °C in 12 h in a pH-stat bioreactor. However, the enzymatic activity at 60 °C was slightly lower than that of LCC^ICCG^. Therefore, the efficiency might not satisfy the requirement for industrial application. Modification of the protein surface charge was also used to mutate the salt-dependent cutinase from *Thermocarpiscus australiensis* (TaC) to salt-free enzymes (Brinch-Pedersen et al. 2024).

Li et al. (2022) designed TfCut2^S121P/D174S/D204P^ with enhanced thermostability by mining molecular dynamics simulation trajectories using machine learning methods. Three mutation sites are in outer loop regions. The variant had the Δ*T*_m_ value of 9.3 °C and showed the increased PET degradation at 70 °C.

### The best performance of engineered thermophilic PET hydrolases

Table 2 shows representative thermophilic PET hydrolases and their mutants. All variants of these PET hydrolases were thermostable above 70 °C for at least 10 h. ThcCut1 (*T*_m_: 72 °C) was mutated by Zhang et al. (2022b) to the best variant ThcCut1^G63A/F210I/D205C/E254C/Q93G^ (ThcCut1^AICCG^; *T*_m_: 92.8 °C), with mutations corresponding to LCC^ICCG^ (Tournier et al. 2020) except for the G63A mutation, which corresponds to the G62A mutation in TfCut2 (Wei et al. 2016). The mutant resulted in the degradation of 96.2% of post-consumer PET bottle particles in 96 h at 70 °C with the addition of dodecyltrimethylammonium bromide. Zeng et al. (2022) improved LCC^ICCG^ by introducing additional mutations: KIP (A59K/V63I/N248P), RIP (A59R/V63I/N248P), and KRP (A59K/V75R/N248P), resulting in higher *T*_m_ values (approximately 99 °C) compared to LCC^ICCG^ (95.2 °C). The optimal temperature for reinforced PET, including 30% glass fiber, was 85 °C. However, their activity against amorphous PET was like that against LCC^ICCG^. In a study by Ding et al. (2023), additional mutations (S32L/D18T/S98R/T157P/E173Q/N213P) were introduced into LCC^ICCG^ (LCC^ICCG^_16M^)^. The mutant exhibited higher efficiency toward crystalline PET powder at 75 °C and toward crystalline PET materials at temperatures between 75 and 80 °C. The mutant digestion of ground crystalline water bottles produced more soluble products than LCC^ICCG^ in 24 h at 75 and 80 °C, resulting in a degradation level of approximately 30%. This means the 70% residues and the effect of recrystallization is not described. LCC-A2, further mutated variant of LCC^ICCG^ (Zheng et al. 2024), displayed higher performance for the pretreated, post-consumer PET waste at 78 °C than at 72 °C, showing > 90% depolymerization of 200 g/kg. Newly cloned Kubu-P was mutated to Kubu-P^M12^ (Kubu-P^T95R/D230S/E131Q/Q127S/D190HA279S/T119N/V184I^), which showed better performance under PET load of 20 and 30% PET at 70 °C, compared to LCC^ICCG^ (Seo et al. 2024). Seo et al. (2024) also suggested the production of BHET (21 mM) by enzymatic glycolysis of PET using Kubu-P^M12^ in ethylene glycol, although the conversion rate is 8% in 48 h at 40 °C. BHET is readily hydrolyzable and detectable at trace level in the rection mixture of PET with PET hydrolases. Production of new materials might be significantly in line with upcycling of degradation products from PET (Amalia et al. 2024; Satta et al. 2024). Complete enzymatic depolymerization of PET produces TPA and ethylene glycol, which can be resynthesized to PET to close a loop from monomer production to regenerate monomers using PET waste (Tournier et al. 2023).

Achieving the 90% conversion required for industrialization at high temperatures above 70 °C can be challenging due to PET recrystallization competing with depolymerization (Arnal et al. 2023). Arnal et al. (2023) reported that LCC^ICCG^ performed better at 68 °C than at 72 °C (Tournier et al. 2020), achieving 98% conversion of amorphized PET within 24 h. Cui et al. (2024) used a computational strategy to redesign BhrPETase into TurboPETase, which reduced its *T*_m_ value from 101 °to 84 °C and acquired the highest activity outperforming LCC^ICCG^. The enzyme displayed nearly complete PET depolymerization at 65 °C with 200 g/kg substrate loading and 2 mg_enzyme/_g_PET_ in 8hr. Under the same conditions, LCC^ICCG^ completed PET depolymerization at 65 °C in 16 h. LCC-A2 displayed the best performance at 78 °C, compared to 72 °C and 81 °C (Zheng et al. 2024). The enzyme nearly completed depolymerization with 200 g/kg substrate loading and 0.6 mg_enzyme/_g_PET_ in 8 h. It remains to be fully elucidated which strategy, preventing recrystallization below 68 °C or rapid depolymerization at 78 °C, is cost-effective and appropriate. PET hydrolases have not been reported for the highly crystalline forms of the polymer, especially those found in consumer products (Erickson et al. 2022; Tournier et al. 2023; Arnal et al. 2023). Amorphization is one way to address the recalcitrance of crystalline PET to enzymatic attacks.

## Engineering of type II enzymes

### Type IIa

PET2 is a thermophilic type IIa hydrolase. The wild-type enzyme has one disulfide bond at the corresponding position of type I enzymes. The *T*_m_ value of the enzyme was increased by 3 °C by introducing an additional disulfide bond at the N-terminal position (R42C-G89C), which differs from the metal-binding site of type I enzymes (Nakamura et al. 2021). *Is*PETase mutants with disulfide bonds have been engineered, resulting in increased thermostability and activity, as described below. Therefore, introducing a disulfide bond at an appropriate position is an effective engineering strategy to enhance the thermostability and activity of type I and II enzymes. Multiple single mutations were identified that exhibited higher *T*_m_ or better activity than wild-type PET2. These mutations were combined to create PET2R47C/G89C/F105R/E110K/S156P/G180A/T297A, which demonstrated a 6.7 °C higher *T*_m_ and three times higher activity at 60 °C than the wild-type.

*Ct*PL from *C. taiwanensis* is a thermophilic type IIa enzyme (Chen et al. 2021). *Ct*PL^H210S/F214Ile/N181A/F235L^, expressed in *Pichia pastoris* (which showed better expression than in *Escherichia coli*), was active at 60 °C (Li et al. 2023). His and Phe in subsite I, which are common to type I enzymes, were mutated to Ser and Ile, respectively, as found in *Is*PETase. Furthermore, Phe, which corresponds to Phe243 in subsite II of LCC, was mutated to Leu, like PHL7 (PES-H1). N181A was used to remove *N*-glycosylation sites and restore the hydrolytic activity of PET.

### Type IIb

Numerous studies have been conducted on mutational enhancements of *Is*PETase. Initially, the research focused on boosting activity at ambient temperature; however, the resulting activities were insufficient for industrial depolymerization. The enzyme’s thermostability was then targeted, as it was discovered that the wild-type enzyme is thermolabile and that enzymatic PET depolymerization is temperature-dependent, as previously stated. Several thermostable *Is*PETases were obtained, as shown in Fig. S2. The first thermostabilization of *Is*PETase through rational protein engineering was reported by Son et al. (2019). *Is*PETase^S121E/D186H/R280A^ (ThermoPETase) increased the *T*_m_ value by 8.81 °C and the activity at 40 °C by 14-fold, compared to the wild-type. Cui et al. (2021) improved the robustness of *Is*PETase using a computational strategy (GRAPE). The mutant enzyme, DuraPETase (*Is*PETaseS121E/I168R/W159H/S188Q/R280A/A180I/G165A/Q119Y/L117F/T140D), exhibited a *T*_m_ value of 77 °C, which is 31 °C higher than the original value. Furthermore, it demonstrated over 300-fold activity toward semicrystalline PET film at mild temperatures (37 °C for 10 days). The W159H mutation altered the Try-Ser pair in subsite II, which is typical of type IIb enzymes, to His in type I enzymes. The Q119Y/L117F mutation resulted in a change in the LCC sequence. Therefore, DuraPETase appears to have acquired the characteristics of type I enzymes.

The introduction of a disulfide bond at the corresponding metal-binding site in type I enzymes can increase the thermostability of *Is*PETase. For example, *Is*PETaseTM^N233C/S282C^ (*Is*PETase^S121E/D186H/R280A/N233C/S282C^) exhibits a *T*_m_ value of 68 °C, which is 23 °C higher than the wild-type. This leads to enhanced thermostability at temperatures up to 60 °C (Brott et al. 2021). Lu et al. (2022a) introduced five mutations in *Is*PETase by machine leaning-aided engineering, designated as FAST-PETase (*Is*PETase^N233K/R224Q/S121E/D186H/R280A^), based on predictions and scaffolds from wild-type *Is*PETase, ThermoPETase, and DuraPETase. In 1 week, FAST-PETase almost completely degraded untreated post-consumer PET at 50 °C. HotPETase, which contains 21 mutations compared to the wild-type, was also identified. Three mutations were derived from the initial protein template, ThermoPETase. An additional disulfide bridge was rationally inserted, and another 16 mutations were identified by directed evolution using automated, high-throughput platform (Bell et al. 2022). The enzyme achieved a *T*_m_ value of 82.5 °C, which is comparable to that of type I enzymes. The enzyme can degrade semicrystalline power, milled bottle-grade PET, PET/polyethylene composite film, and amorphous PET film at 60 °C in 5−24 h (Bell et al. 2022). In the crystal structure of wild-type *Is*PETase, Trp185 was present in three conformations, believed to facilitate substrate binding and catalysis in wild-type (Han et al. 2017; Chen et al. 2021). However, in HotPETase, the residue is present as a single conformer, which creates a new π-stacking interaction with Tyr214, restricting its conformational freedom. Efficient PET decomposition at high temperatures does not require flexible Trp185. Arnal et al. (2023) compared the activities of HotPETase and FAST-PETase with those of LCC^ICCG^ and PES-H1^L92F/Q94Y^. The activities of the two *Is*PETase variants were much less than those of the type I enzymes. Cui et al. (2024) also indicated the same results as Arnal et al. (2023).

Shi et al. (2023) recently discovered a highly effective variant of *Is*PETase, DepoPETase (*Is*PETase^N246D/T88I/D220N/S290P/R260Y/N233K/D186H^) through a novel fluorescence-based high-throughput screening. The variant produced 1407-fold more products toward the amorphous PET film at 50 °C and had a *T*_m_ value of 23.3 °C higher than the wild-type. The enzyme enabled complete degradation of untreated post-consumer PET waste (amorphous). DepoPETase exhibited lower activity at 50 °C for 24 h than FAST-PETase, but showed higher activity for 48 h. However, based on the presented finding, this enzyme may not perform as well as type I enzymes.

By combination of three-directional points (protein yield, activity and durability), Lee et al. (2023) carefully created Z1-PETase (*Is*PETaseS121E/D186H/N246D/S242T/N233C-S282C/P181V/A180V/N132E/R224E/A171C-S193C), which depolymerized > 90% of post-consumer PET powders (crystallinity of approximately 10% and a concentration of 2.5 %) within 24 and 8 h at 40 and 55 °C, respectively. The industrialization of Z1-PETase must be dependent on its capability for high loading of PET powder such as 20% levels for LCC^ICCG^ and TurboPETase.

Mutation results indicated that several amino acids are key targets for mutation (Fig. S3). S121E/D186H/R280A are included in ThermoPETase, HotPETase and FAST-PETase, but R280A is not in Z1-PETase. R280A and D186H are included in DuraPETase and DepoPETase, respectively. D233C/S282C mutation is found in HotPETase and Z1-PETase. N233K is found in FAST-PETase and DepoPETase. Ser214 is mutated to His and Tyr in DuraPETase and HotPETase, respectively. Gln119 is mutated to Tyr and Lys in DuraPETase and HotPETase, respectively.

The wild-type *Is*PETase cannot hydrolyze MHET, which is hydrolyzed by MHETase to TPA and ethylene glycol (Yoshida et al. 2016). MHETase is a feruloyl esterase homolog, which was cloned and characterized (Palm et al. 2019; Knott et al. 2020). Knott et al. (2020) reported a synergetic effect of *Is*PETase and MHETase for PET hydrolysis at 30 °C for 96 h. However, MHET is spontaneously hydrolyzed at high temperatures (Arnal et al. 2023), resulting in that MHET is eventually converted into TPA in enzymatic PET hydrolysis during thermophilic enzymes. In addition, intensified *Is*PETase, such as Z1-PETase, can hydrolyze MHET even at 30 °C (Lee et al. 2023).

## Conclusion and perspective

PET-hydrolyzing enzymes are widely distributed, indicating that PET, particularly amorphous PET, can be hydrolyzed in ecosystems at extremely low rates. However, it is important to avoid creating an illusion for the public or overestimating its role, as biodegradation in the ecosystem, even if possible, cannot match the amount of PET released into the environment. The most crucial aspect is to collect and recycle PET waste as much as possible. Enzymatic PET hydrolysis is a promising technology for ecofriendly PET recycling. Recent research has demonstrated the feasibility of industrial PET biorecycling using PET hydrolases. Currently, uncountable PET-hydrolyzing enzymes have been identified, including type I and type II with variations. However, none of the wild-type enzymes are suitable for industrial applications. To achieve a practical level for industrial biorecycling, enzymes have been mutationally improved, with PET waste pretreatment. Variants created from the representative PET hydrolases and their mutational strategies are listed in Table S4 and S5. Currently, no enzyme can meet the requirements for industrialization without pretreatment of PET waste, such as amorphization and micronization. This is because enzyme efficiency highly depends on the ratio of PET dimensions per enzyme, and the degradability of crystalline PET is significantly lower than amorphous PET. PET waste, including polyolefins, PET, and polystyrene, is often discarded as plastic trash without being sorted by type, except for transparent water bottles, which are collected and recycled. The classification of PET waste based on its crystallinity grade, whether amorphous or crystalline, is practically impossible. Therefore, biorecycling requires a balance between improving enzymes and implementing economical and ecofriendly pretreatments. Some enzymes performed better on untreated or crystalline PET waste than LCC^ICCG^, which is still considered as the standard PET hydrolase. However, the degradation of crystalline PET was much lower than amorphous PET, leaving behind undecomposed remnants, mostly crystalline parts. PET stored at temperatures near *T*_g_ undergoes physical aging (Hutchinson 1995). When post-consumer PET waste was kept at 70 °C, its crystallinity gradually increased and reached a threshold of 20% for the upper limit of biodegradation in approximately 10 h (Pfaff et al. 2022). However, after being kept at 65 °C for 24 h, there was almost no increase in crystallinity (Tournier et al. 2020). The amorphous fraction of PET undergoes physical aging at 70 °C, gradually transforming into rigid amorphous fraction adjacent to the crystalline fraction. This transformation results in limited accessibility to enzymatic hydrolysis, as noted by Wei et al. (2019a). The reaction temperature of LCC^ICCG^ was re-optimized from 72 to 68 °C, possibly due to these risks, as suggested by Arnal et al. (2023). TurboPETase displayed better performance at 65 °C than LCC^ICCG^ (Cui et al. 2024) on a laboratory scale (in a 7.5 L bioreactor). The operational temperature for PET hydrolases must be lower than 68 °C. On the other hand, a new LCC variant, LCC-A2 suggested the best performance at 78 °C in a shorter time than LCC^ICCG^ and TurboPETase (Zheng et al. 2024). Further work is needed to evaluate them scientifically and practically. Erickson et al. (2022) used bioinformatics and machine learning to identify 74 putative thermotolerant PET hydrolases with natural diversity. The three candidate enzymes showed higher hydrolysis rates for the crystalline powder compared to the amorphous powder or film. Furthermore, two of the candidates performed better for amorphous films than for amorphous powders. However, their optimal temperatures range from 30 to 60 °C and their activity levels are lower than those of LCC^ICCG^ at 70 °C. Carbios has announced the industrial use of LCC^ICCG^ for enzymatic recycling (https://www.carbios.com/en/enzymatic-recycling/). PET hydrolases that can operate at temperatures lower than 68 °C for shorter than 24 h or PET hydrolases that can degrade crystalline PET to the same extent as amorphous PET, achieving 98% conversion at 20% PET loading, could replace LCC^ICCG^. TurboPETase and LCC-A2 outperform LCC^ICCG^ on a laboratory scale, but their involvement in the industrial biorecycling of PET is yet to be determined, as the industrialization involves several factors other than PET hydrolase and requires the total process refinement is needed.

Taken together, PET hydrolases applicable to industrial depolymerization of PET need enough thermostability close to the *T*_g_ of PET (below 80 °C) and keep sufficient durability and catalytic efficiency of at least 90% (ideally 95%) conversion against amorphous PET (high loading ~ 200 g/L; preferably higher). LCC^ICCG^ and TurboPETase can be the standard enzymes used for PET biorecycling. It should be noted to use LCC^ICCG^ or TurboPETase under its optimal conditions (pH 8.0 and 65-68 °C) on testing. In numerous studies, the activity of each target enzyme has been compared to that of wild-type LCC (even after the publication of LCC^ICCG^) or LCC^ICCG^ at lower temperatures, such as 30−50 °C. No international scientific consensus has emerged for the evaluation of PET hydrolases (Arnal et al. 2023), which require commercially available or voluntarily provided PET powder samples for testing, as an amorphous film from Goodfellows Cambridge, Ltd. was used as the standard substrate for PET hydrolases in the previous stage of research.

The performance of the resultant mutation appears to depend on wild-type enzymes. For example, although thermostabilized mutants of *Is*PETase have been developed, they are still unsuitable for industrial use (Arnal et al. 2023; Cui et al. 2024). This is likely because PET hydrolases have comparable core structures, but differ in their thermostability and activity levels, which are determined by their unique sequences. Thermostabilized mutants of *Is*PETase have been modified with the same amino acids found in type I enzymes, such as W159H/L117Y/Q119Y (DuraPETase) and a disulfide bond (*Is*PETaseTM^N233C/S282C^, and HotPETase), as described above. Positive indexes for improving type I enzymes include the introduction of an additional disulfide bond at the appropriate position, mostly at the cation-binding site, to increase thermostability. Changing an obstacle amino acid to probable extension of the polymer chain was useful. For example, in Cut190, changing Gln138 to Ala and in LCC, changing Tyr127 to Gly were effective. However, in PES-H1^L92F/Q94Y^, a single mutation was attempted by changing Gln94 to Tyr, but it failed. The substitution of Leu136 to Phe in Cut 190 and Leu93 to Phe in PES-H1^L92F/Q94Y^ (LCC has Phe125 at the same position) likely enhanced the interaction between the amino acid and the polymer, resulting in a π-π interaction between Phe and the aromatic ring of TPA. The combination of L92F and Q94Y in PES-H1^L92F/Q94Y^ may create an aromatic channel along with an aromatic clamp (Trp155 and Phe62) (Pfaff et al. 2022). It has yet to be confirmed whether the rule observed in PES-H1 is applicable to others. However, the new findings with PES-H1 and TurboPETase provide clues for improved performance. PES-H1 has His130 and Leu210 in the conserved pair of His and Phe in subsite II of the type I enzyme. Replacement of Leu with Phe reduced the activity, but replacement with Thr improved the activity (Richter et al. 2023). BhrPETase shares a 94% identity with LCC and has a pair of His and Phe like LCC. However, its efficient mutant, TurboPETase, has a pair of His and Thr (Cui et al. 2024). LCC^ICCG^ contains His and Ile residues instead of Phe. Therefore, the conserved amino acids can be carefully mutated. Leu/Ile and Thr may lead to a more flexible binding groove than that in the wild-type. TurboPETse changed the pair of His218 and F189 at subsite I to Ser and Ile, respectively, which are characteristic of *Is*PETase. This change is believed to contribute to a more flexible binding groove, along with the Phe243 to Thr mutation at subsite II. These changes improve flexibility in subsites I and II, which is considered the fourth index. The upcoming research stage will concentrate on PET hydrolases that can convert higher loadings of PET at lower temperatures, comparable to those of LCC^ICCG^ and TurboPETase (preferably shorter). Furthermore, the focus will be on those that work better on crystalline PET to minimize the PET pretreatment steps. Arnal et al. (2023) suggested that acid-tolerant PET hydrolases, which can efficiently depolymerize PET with no (or minimal) need for soda for pH regulation, would be useful in industrial applications.

The three most efficient templates for PET hydrolase (LCC, PES-H1, and BhrPETase) were identified using a metagenomic approach. This suggests that a metagenome-derived enzyme gene is a mixture of multiple genes and is considered a mutant.

Müller et al. (2005) discovered the first PET hydrolases approximately 20 years ago. Carbios is constructing the first plant for the enzymatic depolymerization of PET in Longlaville, Grand-Est Region, France. The plant is expected to start the first significant deliveries to clients using LCC^ICCG^ in 2026. From the end of 2026, Hündgen Entsorgungs GmbH & Co. KG will supply 15kt/year of post-consumer PET flakes to the first commercial plant (carbios.com/en/enzymatic-recycling/). TurboPETase (Cui et al. 2024) and LCC-A2 (Zheng et al. 2024) outperformed LCC^ICCG^, but the enzyme must overcome barriers of practical application for biorecycling. Further improvement of PET hydrolases would be beneficial for future PET biorecycling. The knowledge accumulated regarding PET hydrolases is also useful for the biorecycling of other polymers.

Now, the main target of biorecycling is transparent or colored bottles, which make up around 30% of PET products and fibers occupy approximately 60 % (Tournier et al. 2023). Packaging films comprising amorphous PET occupy the third volume of all PET products. They are potential targets for biorecycling, when they are separated from other polymer packages such as polyethylene, polypropylene, and polystyrene packages. Polyester fibers are made of semicrystalline PET, which has a higher crystallinity than bottles. They can be targeted for the biorecycling, using the same technology as that for PET bottles. However, fibers are often used in mixed forms with other natural or synthetic materials, and garments need more complex processes to remove accessories such as buttons and fasteners (natural, synthetic, or metal materials). Polymer-recycling is closely linked to social systems such as bans, laws, and waste-collection networks, thereby not dependent only on technological feasibility. However, expansion of the target for the biorecycling must contribute to reducing the plastic release into the environment. Upcycling of degradation products (TPA and ethylene glycol) coupled with biorecycling of PET has been tried (Amalia et al. 2024; Satta et al. 2024), but its future would depend on technological development and economical profitability.

PET is unsusceptible to decomposition by UV, unlike polyolefins, which are prone to UV-induced decomposition and become a constituent component of microplastics. Nevertheless, PET microplastics occupy approximately half of the total microplastic, as they are released in household wastewater through polyester fabric laundry (garments, carpets, textiles etc.). Detergents contain various enzymes (https://biosolutions.novozymes.com/en/dish/insights/article/beginners-guide-enzymes-detergents). PET hydrolases are expected to prevent pilling on the surface of polyester fabrics. They might also offer a solution for removing microplastics and microfibers generated through laundry as one of the detergent enzymes. This is also an industrial application of PET hydrolases. For laundry purpose, PET hydrolases might satisfy new requirements, such as working at moderately high temperatures and accomplishing the high decomposition of pilling and microplastics (far lower concentration of PET materials in washings compared to industrial biorecycling). Micro- and nanoplastics have far broader surface than minimized PET particles for industrial biorecycling, which is beneficial to PET hydrolases. Enzymatic removal of microplastics and microfibers of PET can be technologically possible for municipal wastewater treatment.

## Supplementary information

ESM 1 (PDF 2.43 mb)

## Data Availability

This paper includes no original data except the modeling of enzymes with a model substrate in Fig. 2. The detail of the modeling is shown in Supplementary Information (Scheme S1 and Fig. S4).
