# Supplementary material for: Engineered polyethylene terephthalate hydrolases: perspectives and limits
Source: Appl Microbiol Biotechnol. 2024 Jul 2;108(1):404. doi: 10.1007/s00253-024-13222-2 (PMC11219463; doi:10.1007/s00253-024-13222-2)
Supplement: Supplementary file 1 — (PDF 2.43 mb) [file 253_2024_13222_MOESM1_ESM.pdf]

Minireview for Applied Microbiology and Biotechnology

Supplementary Information

Engineered polyethylene terephthalate hydrolases: perspectives and limits

Fusako Kawai[1*§], Ryo Iizuka[2§] and Takeshi Kawabata[3]

[1] Graduate School of Environmental and Life Sciences, Okayama University, 1-1-1 Tsushima-Naka, Kita-Ku, Okayama 700-8530, Japan

[2] Graduate School of Science, The University of Tokyo, 7-3-1 Hongo, Bunkyo-Ku, Tokyo 113-0033, Japan

[3] Graduate School of Information Sciences, Tohoku University, Aoba 6-3-09, Aoba-ku, Sendai. Miyagi, 980-8579, Japan

[*] Corresponding author: Fusako Kawai E-mail: fkawai@okayama-u.ac.jp

[§] equal contribution

Scheme S1. Modeling procedures of PET hydrolases bound with a PET dimer

PET dimer structures bound to PET hydrolases were constructed using template-based modeling. The structure of TaBTa (1,4-butanediol diterephthalate) bound to $LCC^{ICCG\text{-}S165A}$ (PDB ID: 8JMP) was used as the template ligand molecule. TaBTa is a soluble fragment of poly(butylene adipate-*co*-terephthalate) (PBAT). The modeling comprised four steps. First, the initial 3D structure of a PET dimer was built using ChemDraw 18.2 and Chem3D 18.2. Second, the protein structure of $LCC^{ICCG\text{-}S165A}$ (PDB ID: 8JMP) was superimposed on Cut190 (PDB ID: 4WFK) and PES-H1 (PDB ID:7CUV) using Matras 13.0 (Kawabata and Nishikawa 2000). The TaBTa ligand structure (comp_id: EMX) in 8JMP was also transposed into the Cut190 and PES-H1 structures. Third, the structure of the PET dimer was flexibly superimposed onto the transposed TaBTa molecule using the *fkcombu* program (Kawabata and Nakamura 2014). Atom matching was decided by the topologically constrained disconnected maximum common substructure (TD-MCS) with θ=2 (Kawabata 2011), as shown in Fig. S3. Finally, energy minimization using the program AMBER 20 (Case et al. 2021) to refine the 3D complex models of the protein and ligand molecules was generated by template modeling. We used the GBSA implicit solvent model with the ff19SB and gaff2 force fields. Energy minimization was performed with positional restraints on the protein-heavy atoms. The force field parameters for the ligand were set according to a tutorial page (https://ambermed.org/tutorials/basic/tutorial4b).

The mutated structures, $Cut190^{L136FQ138A}$ and $PES\text{-}H1^{L92FQ94F}$, were modeled using the Rotamer tool of UCSF Chimera 1.15 (Pettersen et al. 2004). The rotamers with the highest probabilities were selected, except for Q94Y in $PES\text{-}H1^{L92FQ94F}$. For Tyr at the 94th site of PES-H1, the rotamer with the 4th highest probability was chosen because the top three rotamers with the highest probability had serious atomic clashes with the transposed TaBTa structure.

Table S1. PET hydrolases used for constructing a phylogenetic tree

Enzyme	Type	Source organism	GenBank AC / PDB ID
**Fungi**			
FsC	Fungal type I	*Fusarium solani* (currently *F. vanettenii)*	PDB_ID:1OXM
HiC	Fungal type I	*Humicola insolens*	PDB_ID:4OYY
TaC	Fungal type I	*Thermocarpiscus australiensis*	Not deposited[1)]
AfC	Fungal type I	*Aspergillus fumigatiaffinms*	PDB_ID:8JCT
**Actinomycetes**			
BTA1	Type I	*Thermobifida fusca* DSM43793	AJ810119.1
TfCut2	Type I	*T. fusca* KW3	CBY05530.1
Thc_cut2	Type I	*T. cellusilytica* DSM44535	ADV92527.1
Est119	Type I	*T. alba* AHK119	BAK48590.1
Thh_Est	Type I	*T. halotolerans* DSM44931	AFA45122.1
Tcur1278	Type I	*Thermomonospora curvata* DSM43183	CDN67545.1
Cut190	Type I	*Saccharomonospora viridis* AHK190	BAO42836.1
CaPETase	Type I	*Cryptosporangium aureantiacum*	SHM40309.1
Mipa-P	-	*Micromonospora pattaloongensis* DSM45254	Not deposited[2)]
Kubu-P	-	*Kutzneria buriramensis* DSM 45791	Not deposited[2)]
503	Type I	*Nocardioidaceae* bacterium Broad-1	EGD44994.1
611	Type I	*Saccharopolyspora flava*	WP_093412886.1
**Metagenome**			
LCC	Type I		AEV21261.1
BhrPETase	Type I	bacterium HR29	GBD22443.1
PES-H1	Type I		PDB_ID: 7CUV LT571446.1
**Bacteria other than actinomycetes**			
DmPETase	Type I	*Deinococcus maricopensis* DSM 21211	ADV66860.1
Cbotu_EstA	-	*Clostridium botulinum* ATCC 3502	KP859619.1
PET2	Type IIa	Uncultured bacterium	ACC95208.1
PET6	Type IIb	*Vibrio gazogenes*	WP_021018894.1
PET27	Type IIb	*Aequorivita* sp. CIP111184	WP_111881932.1
PET30	Type IIb	*Kaistella* (*Chryseobacterium*) *jeonii*	WP_039353427.1
CtPL	Type IIa	*Caldimonas taiwanensis*	WP_062195544.1
*Is*PETase	Type IIb	*Ideonella* (*Piscinibacter*) *sakaiensis*	GAP38373.1

BurPL	Type IIb	*Burkholderiales* bacterium	OGB27210.1
Mors1	Type IIa	*Moraxella* sp. TA144	CAA37220.1
PE-H	Type IIa	*Pseudomonas aestusnigri*	WP_088276085.1
**Others**			
MG8	Type IIa	Human saliva	Not deposited[3)]

[1)] Brinch-Pedersen et al. 2024
[2)] Seo et al. 2024
[3)] Eiamthong et al. 2022

Table S2. Representative type I PET hydrolases

**Enzyme**	**Source**	**GenBank accession**	**UniProt accession**	**Reference**	**PDB ID**	**Reference**
LCC	Metagenome from leaf-branch compost	HQ704839	G9BY57	Sulaiman et al. 2012	4EB0 (Wild-type)	Sulaiman et al. 2014
					6THS (S165A)	Tournier et al. 2020
					6THT (ICCG[a]/S165A)	
					7VVC (ICCG[a]/S165A)	Zeng et al. 2022
					7VVE (ICCG[a]/S165A, MHET-bound)	
					7W1N (KRP[b])	
					7W44 (RIP[c])	
					7W45 (KIP[d])	
					8JMO (ICCG[a]/S165A, BTa-bound)	Yang et al. 2024
					8JMP (ICCG[a]/S165A, TaBTa-bound)	
BhrPETase	bacterium HR29	BEIF01000001	A0A2H5Z9R5	Xi et al. 2021	7EOA (Wild-type)	Unpublished
TfCut2	*Thermobifida fusca* KW3	FR727681	Q6A0I4	Herrero Acero et al. 2011	4CG1 (S58R/T176S)	Roth et al. 2014
		HG939556			4CG2 (S58R/T176S, PMS-bound)	
					4CG3 (S58S/T176S)	
					5ZOA (Wild-type)	Unpublished
					7QJQ (S58S/N88S/R268W)	Erickson et al. 2022
					7QJR (S58R/T176S/T205R/A222L/K226R/A234T)	
					7XTR (S58R/T176S/H224S/F228I)	Yang et al. 2023
					7XTS (S58R/S170A/T176S/H224S/F228I)	
					7XTT (S58R/S170A/T176S/H224S/F228I, MHET-bound)	
					7XTU (S58R/S170A/T176S)	
					7XTV (S58R/S170A/T176S, MHET-bound)	
ThcCut1	*Thermobifida cellulosilytica* DSM44535	HQ147785.1	E9LVH8	Herrero Acero et al. 2011	5LUI (Wild-type)	Ribitsch et al. 2017
Cut190	*Saccharomonospora viridis* AHK190	AB728484	W0TJ64	Kawai et al. 2014	4WFI (S226P, Ca^{2+}-free)	Miyakawa et al. 2015
					4WFJ (S226P, Ca^{2+}-bound)	
					4WFK (S226P, Ca^{2+}-bound)	
					5ZNO (S176A/S226P/R228S, Ca^{2+}-bound)	Numoto et al. 2018
					5ZRQ (S176A/S226P/R228S, Zn^{2+}-bound)	
					5ZRR (S176A/S226P/R228S, MES-bound)	

					5ZRS (S176A/S226P/R228S, MEA-bound)<br>7CEF (Cut190**[e])<br>7CEH (Cut190**[e]76A)<br>7CTR (Cut190*SS[f])<br>7CTS (Cut190*SS[f]176A)<br>8IBL (Cut190*SS[f]S176A, MES-bound)<br>8IBM (Cut190*SS[f]S176A, sulfate-bound)	 Senga et al. 2021<br><br>Emori et al. 2021<br><br>Numoto et al. 2023
PHL7<br>/ PES-H1	Metagenome from compost	LT571446		Sonnendecker et al. 2022<br>Pfaff et al. 2022	7NEI (Wild-type)<br><br>7CUV (Wild-type)<br>7E30 (Wild-type, Citrate-bound)<br>7W6C (Wild-type, MHETA-bound)<br>7W6O (Wil- type, MHETA-bound)<br>7W6Q (Wild-type, MHETA-bound)	Sonnendecker et al. 2022<br>Pfaff et al. 2022

[a] ICCG: Y127G/D238C/F243I/S283C

[b] KRP: ICCG-A59K/V75R/N248P

[c] RIP: ICCG-A59R/V63I/N248P

[d] KIP: ICCG-A59K/V63I/N248P

[e] Cut190**: S226P/R228S/K305del/L306del/N307del

[f] Cut190*SS: S226P/R228S/Q138A/D250C/E296C/Q123H/N202H

MHET: mono-(2-hydroxyethyl) terephthalate

BTa: 4-((4-hydroxybutoxy)carbonyl)benzoic acid

TaBTa: 1,4-butanediol diterephthalate
PMS: phenylmethanesulfonic acid
MES: monoethyl succinate
MEA: monoethyl agipate
MHETA: 4-(2-hydroxyethylcarbamoyl) benzoic acid

Table S3. Representative type II PET hydrolases

**Enzyme**	**Type**	**Source**	**GenBank accession**	**Uniprot accession**	**Reference**	**PDB ID**	**Reference**
LipIAF5.2 /PET2	IIa	Metagenome from the biomass collected from a gelatin-enriched fed-batch reactor	EU660533<br>ON416993	C3RYL0	Meilleur et al. 2009<br>Danso et al. 2018	7EC8 (PET M2)<br>7ECB (PET M7)	Nakamura et al. 2021
*Pa*PETase /PE-H	IIa	*Halopseudomonas aestusnigri* (*Pseudomonas aestusnigri*)	OWL88088	A0A1H6AD45	Bollinger et al. 2020	6SBN (Wild-type)<br>6SCD (Y250S)	Bollinger et al. 2020
PET6	IIa	*Vibrio gazogenes*	WP_077316261	A0A1M5F0K3	Weigert et al. 2022	7Z6B (Wil-type)	Weigert et al. 2022
*Ct*PL	IIa	*Caldimonas taiwanensis*	WP_062195544	-	Chen et al. 2021	8IAN (*Ct*PL-DM)<br>8IBI (*Ct*PL-DM-S155A)<br>8IBJ (*Ct*PL-DM/N181A/F235L/S155A)	Li et al. 2023
*Is*PETase	IIb	*Ideonella* (*Piscinibacter*) *sakaiensis* 201-F6	WP_054022242	A0A0K8P6T7	Yoshida et al. 2016	5XFY (S131A)<br>5XFZ (R103G/S131A)<br>5XG0 (Wild-type)<br>5XH2 (R103G/S131A, pNP-bound)<br>5XH3 (R103G/S131A, HEMT-bound)<br>5XJH (Wild-type)<br>5YFE (T72N/R224A/A287N)<br>5YNS (R280A)<br>6ANE (Wil-type)<br>6EQD-H (Wild-type)<br>6IJ3 (S121D/D186H)<br>6IJ4 (S121E/D186H)<br>6IJ5 (P181A)<br>6IJ6 (**ThermoPETase**)<br>6ILW (Wild-type)<br>6ILX (W159F)<br>6KUO (N246D)<br>6KUQ (A248D/R280K)<br>6KUS (S121E/D186H/S242T/N246D)<br>6KY5 (**DuraPETase**)	Han et al. 2017<br><br><br><br><br>Joo et al. 2018<br>Liu et al. 2018<br><br>Fecker et al. 2018<br>Austin et al. 2018<br>Son et al. 2019<br><br><br><br>Liu et al. 2019<br><br>Unpublished<br><br><br>Cui et al. 2021

						6QGC (Wild-type)<br>7CQB (N233A)<br>7CY0 (S185H)<br>7OSB (S238F/W159H)<br>7QVH (**HotPETase**)<br>7SH6 (**FAST-PETase**)<br>7VWN (T29S/K95N/I168R/P181V/S214V/N233C/A248D/R280A/S282C)<br>7XTW (R132G/S160A, MHET-bound)<br>8CRU (GrAnc8)<br>8D1D (PROSS5)<br>8GU4 (With linker)<br>8GU5 (Wild-type)<br>8J17 (S121P/D186A)<br>8J45 (S121E/D186H/R224Q/N233K/R280A)<br>8J5N (V14E/L18P/R53Q/V84L/D186H/F201I/F229Y/N233K/R280E/D283R)<br>8H5M (Is-4pC)<br>8H5O (Is-4pC+P181V)<br>8H5J (Is-8p)<br>8H5K (**Z1-PETase**)<br>8H5L (Z1-PETase+ A202C/V211C/S214Y/N275C/F284C)<br>8H83 (V14E/L18P/V84L/F201I/E204Q/F229Y/N233K/R280E/D283R)	Palm et al. 2019<br>Unpublished<br>Chen et al. 2021<br>Erickson et al. 2022<br>Bell et al. 2022<br>Lu et al. 2022<br>Unpublished<br>Yang et al. 2023<br>Joho et al. 2023<br>Unpublished<br>Chen et al. 2022<br>Yin et al. 2024<br>Chen et al. 2024<br>Unpublished<br>Lee et al. 2023<br>Unpublished
PET12 /*Pb*PL	IIb	*Caldimonas brevitalea* DSM 7029 (*Polyangium brachysporum* DSM 7029)	WP_047194864	A0A0G3BI90	Danso et al. 2018<br>Chen et al. 2021		
*Bur*PL	IIb	*Burkholderiales* bacterium	OGB27210	A0A1F4JXW8	Chen et al. 2021	7CWQ (Wild-type)	Chen et al. 2021

PET2 M2: F105R/E110K
PET2 M7: R47C/G89C/F105R/E110K/S156P/G180A/T297P
*Ct*PL-DM: H210S/F214I
ThermoPETase: S121E/D186H/R280A

DuraPETase: L117F/Q119Y/T140D/W159H/G165A/I168R/A180I/S188Q/S214H/R280A

HotPETase: S58A/S61V/R90T/K95N/Q119K/S121E/M154G/P181V/Q182M/D186H/S207R/N212K/S213E/S214Y/R224L/N233C/N241C/K252M/T270Q/R280A/S282C

FAST-PETase: S121E/D186H/R224Q/N233K/R280A

GrAnc8: T29S/A33Q/N37D/A40T/A41S/A47R/T51S/R53A/T77K/K95N/A135V/N138S/G147N/A152S/M154L/A171R/D186N/T189K/F201I/S207T/S214H/I218F/D220N/A226P/F229Y/T270S/E274S/T279S/R280S/A287E

PROSS5: Y32H/A33M/R90T/S125D/R132D/V134L/G139N/T140D/A152S/Q182M/D186S/S214H/D220N/T270Q/R280A

Is-4pC: S121E/D186H/N233C/S242T/N246D/S282C

Is-4pC+P181V: S121E/P181V/D186H/N233C/S242T/N246D/S282C

Is-8p: S121E/A180V/P181V/D186H/N233C/S242T/N246D/S282C

Z1-PETase (Is-8p^{Com6}): N37D/S121E/R132E/A171C/A180V/P181V/D186H/S193C/R224E/N233C/S242T/N246D/S282C

pNT: *p*-nitrophenol

HEMT: 1-(2-hydroxyethyl) 4-methyl terephthalate

MHET: mono-(2-hydroxyethyl) terephthalate

Table S4. PET hydrolases and their mutans

Type	Enzyme	T_m [°C]	Degradation properties	Reference
I	LCC			
	Wild-type	86.2		Sulaiman et al. 2014
	LCC^{ICCG}	94.0	Depolymerized 90% of micronized post-consumer colored-flake PET waste in less than 10 h at 72 °C	Tournier et al. 2020
	LCC^{WCCG}	98.0	Depolymerized 90% of micronized post-consumer colored-flake PET waste in less than 10 h at 72 °C	Tournier et al. 2020
	RIP	98.0	Exhibited higher efficacy than LCC^{ICCG} in depolymerizing both amorphous and crystalline PET at 74 °C	Zeng et al., 2022
	KIP	98.9	Exhibited higher efficacy than LCC^{ICCG} in depolymerizing both amorphous and crystalline PET at 74 °C	Zeng et al., 2022
	KRP	98.6	Exhibited higher efficacy than LCC^{ICCG} in depolymerizing both amorphous and crystalline PET at 74 °C	Zeng et al., 2022
	$LCC^{S101N/F243T}$	76.6	Depolymerized fully 1.3 g of untreated post-consumer PET waste in ≤ 3 days at 55 °C	Pirillo et al. 2023
	LCC^{ICCG}_16M	-		Ding et al. 2023
	LCC-A2	95.3	Depolymerized >90% of the pretreated, post-consumer PET waste within 3.3 h at 78 °C	Zheng et al. 2024
I	BhrPETase			
	Wild-type	101		Xi et al. 2021
	TurboPETase	84	Depolymerized post-consumer PET bottles nearly completely in 8 h at a high substrate loading at 65 °C	Cui et al. 2024
I	Cut190			
	Wild-type	70.6*		Kawai et al. 2014
	Cut190**SS/L136F/Q138G	84.7**		Kawai et al. 2023
I	TfCut2			
	Wild-type	71.4		Li et al. 2022
	G62A/F209A	-		Furukawa et al. 2019
	G62A/F209I/E249R	-		Mrigwani et al. 2022
	S121P/D174S/D204P	80.7		Li et al. 2022
	D204C/E253C/H184S/Q92G/F209I/I213K	-		Chen et al. 2022
I	ThcCut1			
	Wild-type	72		Zhang et al. (2022)
	$ThcCut1^{AICCG}$	92.8	Degraded 96.2% of the post-consumer PET bottle particles within 96 h at 70 °C	Zhang et al. (2022)
	PHL7/PES-H1			
	Wild-type	79.1		Sonnendecker et al. 2022
	$PES\text{-}H1^{L92F/Q94Y}$	ΔT_m: 6.4		Pfaff et al. 2022
	$PES\text{-}S1^{L210T}$	ΔT_m: 1.36		Richter et al. 2023

I	*Ca*PETase			
	Wild-type	66.8		Hong et al. 2023
	*Ca*PETase^{M9}	83.2	Almost completely decomposed transparent and colored post-consumer PET powder at 55 °C within half a day	Hong et al. 2023
I	Kubu-P			
	Wild-type	92.8		Seo et al. 2024
	Kubu-P^{M12}	Over 99.9	Better depolymerization of ~30% PET load at 70 °C than LCC^{ICCG}	
IIa	PET2			
	Wild-type	69.0		Danso et al. 2018
	PET2 7M	75.7		Nakamura et al. 2021
IIa	*Ct*PL			
	Wild-type	-		Chen et al. 2021
	*Ct*PL-DM^{F235L}	-		Li et al. 2023
IIb	*Is*PETase			
	Wild-type	46.8		Joo et al. 2018
	ThermoPETase	57.6		Son et al. 2019
	DuraPETase	77		Cui et al. 2021
	FAST-PETase	67.1		Lu et al. 2022
	HotPETase	82.5		Bell et al. 2022
	DepoPETase	69.4		Shi et al. 2023
	Z1-PETase	74		Lee et al. 2023

*In the presence of 300 mM Ca^{2+}
**In the presence of 2.5 mM Ca^{2+}

Table S5. A list of mutational strategies

**I. Introduction of disulfide bond(s) increasing activity and thermostability**		
LCC^{ICCG} and LCC^{WCCG}	An additional disulfide bond (D238C-S283C)	Tournier et al. 2020
TurboPETase	An additional disulfide bond (A251C-A281C)	Cui et al. 2024
TfCut2	An additional disulfide bond (D204C-E253C)	Then et al. 2016
ThcCut1 ^{AICCG}	An additional disulfide bond (D205C-E254C)	Zhang et al. 2022
Cut190	An additional disulfide bond (D250C-E296C)	Oda et al. 2018
*Ca*PETase^{M9}	Two additional disulfide bonds (L180C-A202C, R242C-S291C)	Hong et al. 2023
PET2	An additional disulfide bond (R47C-G89C)	Nakamura et al. 2021
HotPETase	An additional disulfide bond (N233C-S282C)	Bell et al. 2022
Z1-PETase	Two additional disulfide bonds (A171C-S193C, N233C-S282C)	Lee et al. 2023
**II. Mutations in a substrate-binding groove**		
A. Mutations in subsite I		
1) Equivalent to Q138 (Cut190) or Y 127(LCC)		
Cut190	Q138A	Oda et al. 2018
LCC^{ICCG} and LCC^{WCCG}	Y127G	Tournier et al. 2023
ThcCut1 ^{AICCG}	Q93G	Zhang et al. 2022
PES-H1 $^{L92F/Q94Y}$	Q94Y	Pfaff et al. 2022
Dura PETase	Q119Y	Cui et al. 2021
HotPETase	Q119K	Bell et al. 2022
2) Equivalent to L136 (Cut190) or F125 (LCC)		
Cut190	L136F	Kawai et al. 2022
PES-H1$^{L92F/Q94Y}$	L92F	Phaff et al. 2022
DuraPETase	L117F	Cui et al. 2021
3) S214 in *Is*PETase to His or Tyr to avoid W185 wobbling		
DuraPETase	S214H	Cui et al. 2021

HotPETase	S214Y	Bell et al. 2022
4) Equivalent to H218 and F222 in LCC to increase the flexibility of the substrate-binding groove		
*Ct*PL-DM	H210S	Li et al. 2023
TurboPETase	F222I	Cui et al. 2024
B. Mutations in subsite II		
1) Equivalent to F243 in LCC to increase the flexibility of the substrate-binding groove		
LCC^{ICCG} and LCC^{WCCG}	F243I/F243W	Tournier et al. 2020
ThcCut1^{AICCG}	F210I	Zhang et al. 2022
PES-1^{L210T}	L210T	Richter et al. 2023
TurboPETase	F243T	Cui et al. 2024
*Ct*PL-DM^{F253L}	F243L	Li et al. 2023
TfCut2$^{G62A/F209A}$<br>TfCut2$^{G62A/F209I}$	F209A<br>F209I	Furukawa et al. 2019;<br>Mrigwani et al. 2022
2) Restricting W159 wobbling in *Is*PETase		
DuraPETase	W159H	Cui et al. 2021
3) Equivalent to T96 in LCC or T88 in *Is*PETase		
DepoPETase	T88I	Shi et al. 2023
C. Combination of mutations in subsite I and subsite II		
TurboPETase	H218S/F222I (subsite I)<br>F243T (subsite II)	Cui et al. 2024
Tfusca$^{D204C/E253C/H184S/Q92G/F209I/I213K}$	H184S/Q92G (subsite I)<br>F209I (subsite II)	Chen et al. 2022
D. Mutations in loop region		
LCC^{ICCG}-KIP, KRP and RIP	N248P	Zeng et al. 2022
LCC-A2	N248D	Zheng et al. 2024
Tfusca$^{D204C/E253C/H184S/Q92G/F209I/I213K}$	I213K	Chen et al. 2022
DepoPETase	N246D	Shi et al. 2023
TfCut2$^{S121P/D174S/D204P}$	S121P/D174D/D204P	Li et al. 2023
Z1-PETase	S242T/N246D	Lee et al. 2023
**III. Removal of product inhibition**		
TfCut2^{G62A}	G62A	Wei et al. 2016
TfCut2^{G62AF209A}	G62A	Furukawa et al. 2019
ThcCut1^{AICCG}	G63A	Zhang et al. 2022
**IV. Change of the surface charge: negative to neutral/acidic**		
*Ca*PETase^{M9}	N109A/V129T/A155R/G196T/R198K	Hong et al. 2023

TaC	E68G/D82A	Brinch-Peterson et al. 2024

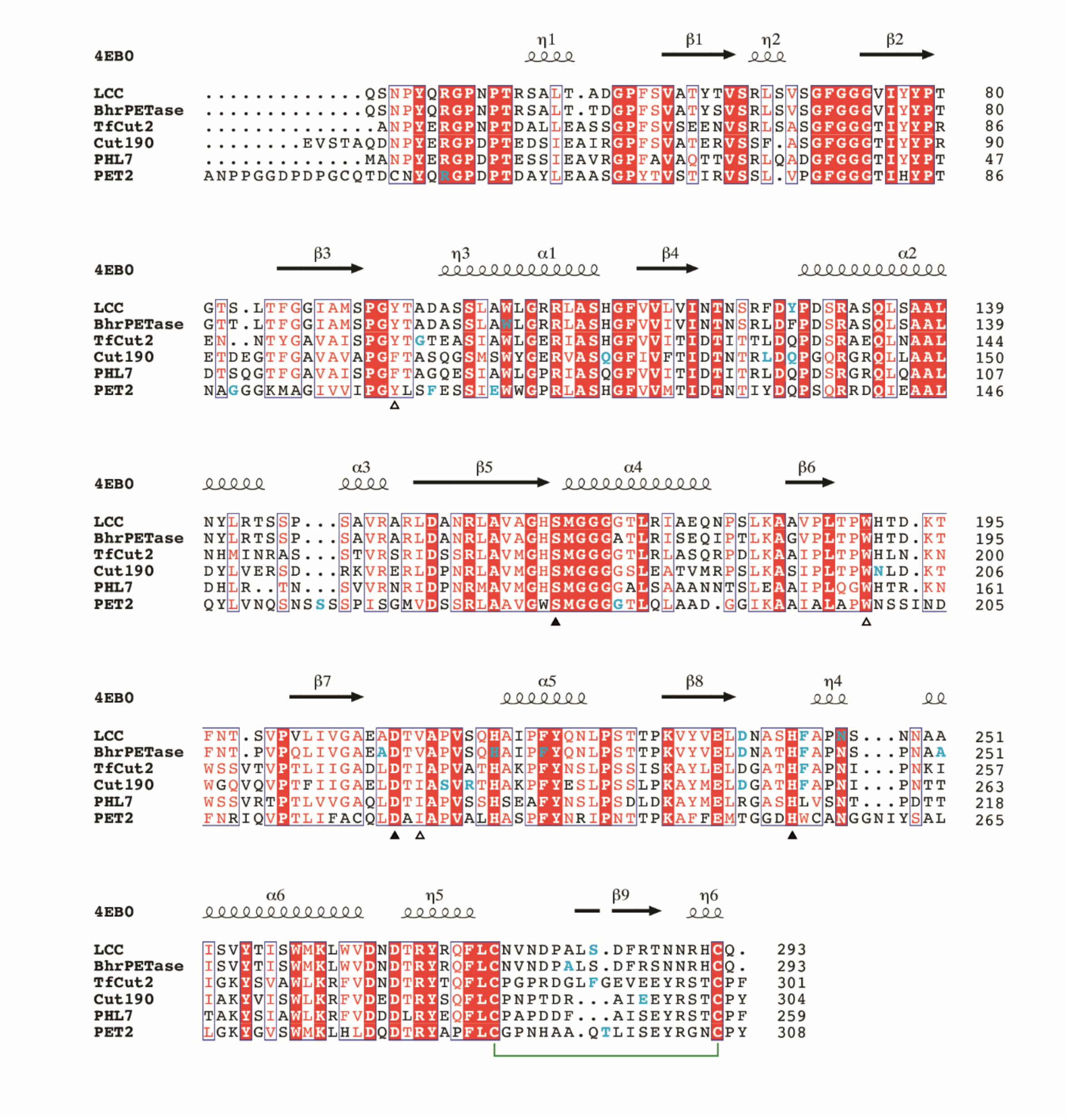

Figure S1. Multiple sequence alignment of type I PET hydrolases

Amino acid sequences of mature type I PET hydrolases (without signal peptides) were aligned using Clustal Omega (https://www.ebi.ac.uk/Tools/msa/clustalo/). The resulting alignment was displayed using ESPript 3.0 (https://espript.ibcp.fr/ESPript/ESPript/) (Robert and Gouet 2014). Residue numbering, including that of the signal peptide, is shown at the right of the sequence. Secondary structure elements are presented based on the crystal structure of LCC (PDB ID: 4EB0) (Sulaiman et al. 2014). The conserved residues are highlighted in boxes. Identical residues are marked with a red background, and highly conserved residues are shown in a red font. Catalytic triads and aromatic clumps are indicated by black and white arrowheads, respectively. Mutated residues are indicated in blue. The green line represents a pair of cysteine residues

forming a disulfide bond. The protein accession numbers were as follows: LCC, HQ704839; PHL7, LT571446; TfCut2, AJ810119; Cut190, AB728484; BhrPETase, GBD22443; and PET2, ON416993.

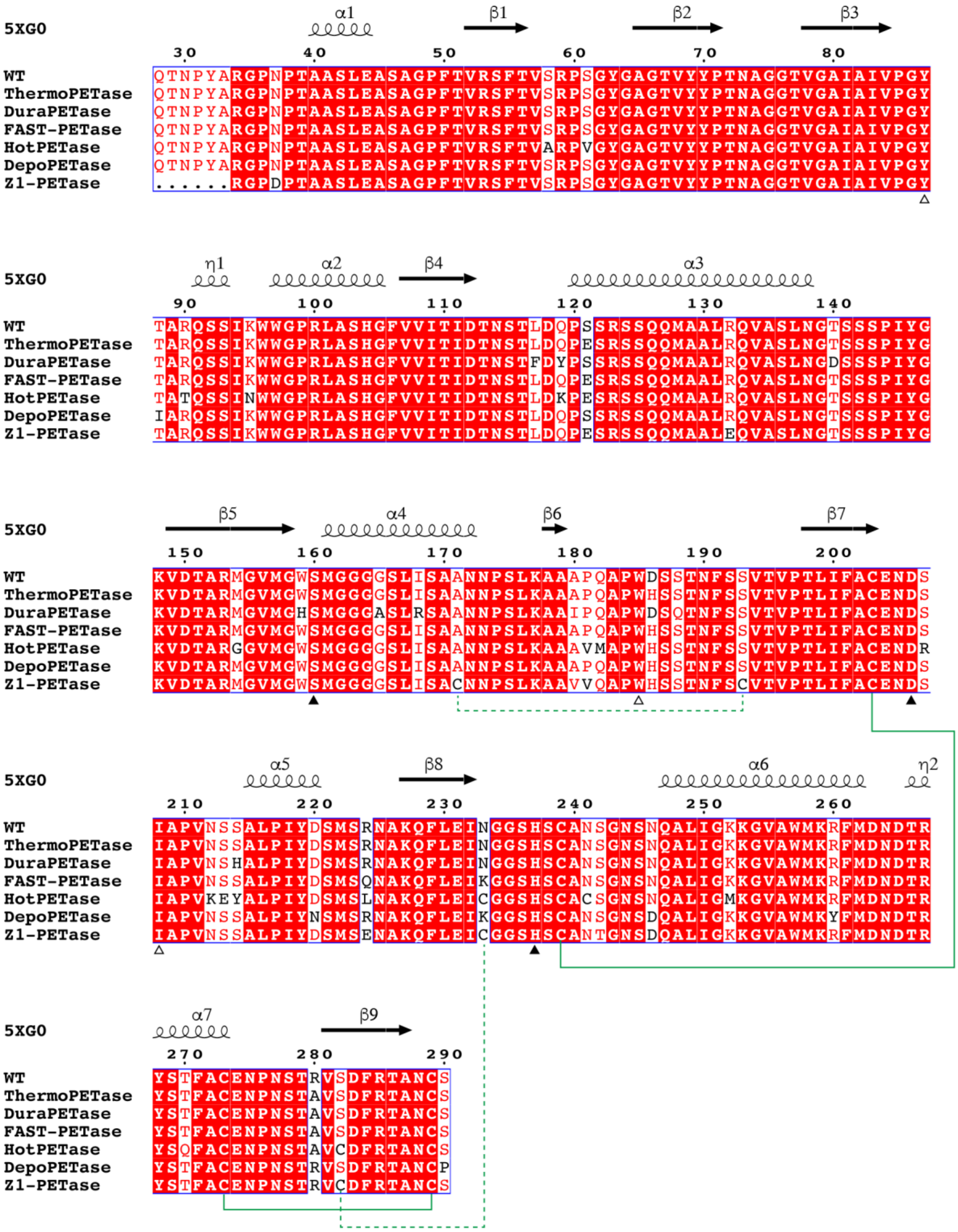

Figure S2. Multiple sequence alignment of *Is*PETase and its variants

The amino acid sequences of mature *Is*PETase and its variants (without signal peptides) were aligned using Clustal Omega. The resulting alignment was displayed using ESPript 3.0 (Robert and Gouet 2014). Residue numbering, including that of the signal peptide, is shown at the top of the sequence. Secondary structure

elements are presented based on the crystal structure of wild-type *Is*PETase (WT; PDB ID: 5XG0) (Han et al. 2017). The conserved residues are highlighted in boxes. Identical residues are marked with a red background, and highly conserved residues are shown in a red font. Catalytic triads and aromatic clumps are indicated by black and white arrowheads, respectively. Green lines represent pairs of cysteine residues that form disulfide bonds. The broken green line represents the third and fourth disulfide bonds introduced into HotPETase and Z1-PETase. The protein accession numbers are as follows: WT, A0A0K8P6T7; ThermoPETase, 6IJ6; DuraPETase, 6KY5; FAST-PETase, 7SH6; HotPETase, 7QVH; and Z1-PETase, 8H5K.

A

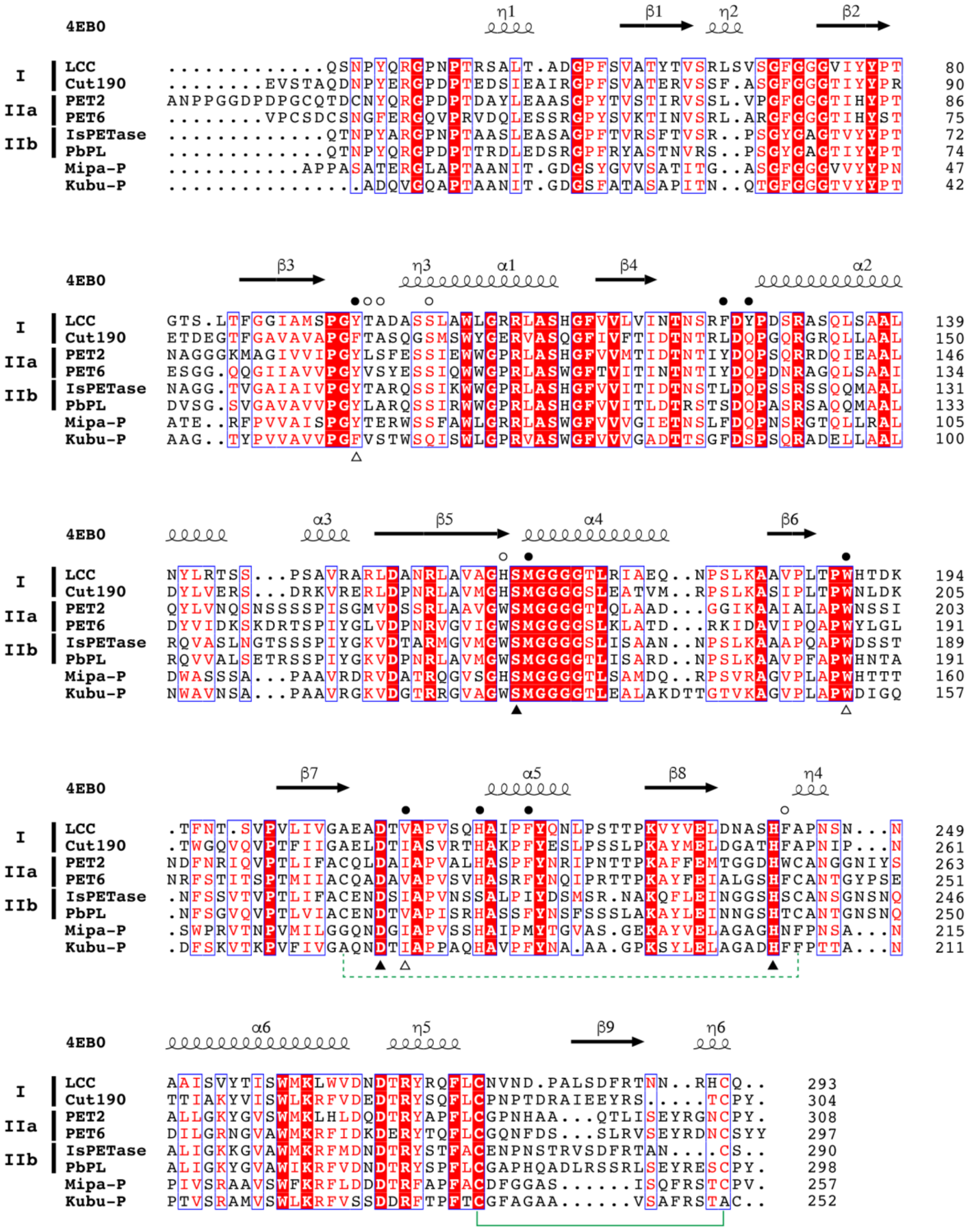
4EB0
η1 β1 η2 β2
I LCC .............QSNPYQRGPNPTRSALT.ADGPFSVATYTVSRLSVSGFGGGVIYYPT 80
Cut190 ........EVSTAQDNPYERGPDPTEDSIEAIRGPFSVATERVSSF.ASGFGGGTIYYPR 90
IIa PET2 ANPPGGDPDPGCQTDCNYQRGPDPTDAYLEAASGPYTVSTIRVSSL.VPGFGGGTIHYPT 86
PET6 ........VPCSDCSNGFERGQVPRVDQLESSRGPYSVKTINVSRL.ARGFGGGTIHYST 75
IIb IsPETase .............QTNPYARGPNPTAASLEASAGPFTVRSFTVSR..PSGYGAGTVYYPT 72
PbPL .............QTNPYQRGPDPTTRDLEDSRGPFRYASTNVRS..PSGYGAGTIYYPT 74
Mipa-P ...........APPASATERGLAPTAANIT.GDGSYGVVSATITG..ASGFGGGVVYYPN 47
Kubu-P ..............ADQVGQAPTAANIT.GDGSFATASAPITN..QTGFGGGTVYYPT 42
4EB0
β3 η3 α1 β4 α2
I LCC GTS.LTFGGIAMSPGYTADASSLAWLGRRLASHGFVVLVINTNSRFDYPDSRASQLSAAL 139
Cut190 ETDEGTFGAVAVAPGFTASQGSMSWYGERVASQGFIVFTIDTNTRLDQPGQRGRQLLAAL 150
IIa PET2 NAGGGKMAGIVVIPGYLSFESSIEWWGPRLASHGFVVMTIDTNTIYDQPSQRRDQIEAAL 146
PET6 ESGG.QQGIIAVVPGYVSYESSIQWWGPRLASWGFTVITINTNTIYDQPDNRAGQLSAAI 134
IIb IsPETase NAGG.TVGAIAIVPGYTARQSSIKWWGPRLASHGFVVITIDTNSTLDQPSSRSSQQMAAL 131
PbPL DVSG.SVGAVAVVPGYLARQSSIRWWGPRLASHGFVVITLDTRSTSDQPASRSAQQMAAL 133
Mipa-P ATE..RFPVVAISPGYTERWSSFAWLGRRLASWGFVVVGIETNSLFDQPNSRGTQLLRAL 105
Kubu-P AAG..TYPVVAVVPGFVSTWSQISWLGPRVASWGFVVVGADTTSGFDSPSQRADELLAAL 100
4EB0
α3 β5 α4 β6
I LCC NYLRTSS...PSAVRARLDANRLAVAGHSMGGGGTLRIAEQ..NPSLKAAVPLTPWHTDK 194
Cut190 DYLVERS...DRKVRERLDPNRLAVMGHSMGGGGSLEATVM..RPSLKASIPLTPWNLDK 205
IIa PET2 QYLVNQSNSSSSPISGMVDSSRLAAVGWSMGGGGTLQLAAD...GGIKAAIALAPWNSSI 203
PET6 DYVIDKSKDRTSPIYGLVDPNRVGVIGWSMGGGGSLKLATD...RKIDAVIPQAPWYLGL 191
IIb IsPETase RQVASLNGTSSSPIYGKVDTARMGVMGWSMGGGGSLISAAN..NPSLKAAAPQAPWDSST 189
PbPL RQVVALSETRSSPIYGKVDPNRLAVMGWSMGGGGTLISARD..NPSLKAAVPFAPWHNTA 191
Mipa-P DWASSSA...PAAVRDRVDATRQGVSGHSMGGGGTLSAMDQ..RPSVRAGVPLAPWHTTT 160
Kubu-P NWAVNSA...PAAVRGKVDGTRRGVAGWSMGGGGTLEALAKDTTGTVKAGVPLAPWDIGQ 157
4EB0
β7 α5 β8 η4
I LCC .TFNT.SVPVLIVGAEADTVAPVSQHAIPFYQNLPSTTPKVYVELDNASHFAPNSN...N 249
Cut190 .TWGQVQVPTFIIGAELDTIASVRTHAKPFYESLPSSLPKAYMELDGATHFAPNIP...N 261
IIa PET2 NDFNRIQVPTLIFACQLDAIAPVALHASPFYNRIPNTTPKAFFEMTGGDHWCANGGNIYS 263
PET6 NRFSTITSPTMIIACQADAVAPVSVHASRFYNQIPRTTPKAYFEIALGSHFCANTGYPSE 251
IIb IsPETase .NFSSVTVPTLIFACENDSIAPVNSSALPIYDSMSR.NAKQFLEINGGSHSCANSGNSNQ 246
PbPL .NFSGVQVPTLVIACENDTVAPISRHASSFYNSFSSSLAKAYLEINNGSHTCANTGNSNQ 250
Mipa-P .SWPRVTNPVMILGGQNDGIAPVSSHAIPMYTGVAS.GEKAYVELAGAGHNFPNSA...N 215
Kubu-P .DFSKVTKPVFIVGAQNDTIAPPAQHAVPFYNA.AA.GPKSYLELAGADHFFPTTA...N 211
4EB0
α6 η5 β9 η6
I LCC AAISVYTISWMKLWVDNDTRYRQFLCNVND.PALSDFRTNN..RHCQ.. 293
Cut190 TTIAKYVISWLKRFVDEDTRYSQFLCPNPTDRAIEEYRS.....TCPY. 304
IIa PET2 ALLGKYGVSWMKLHLDQDTRYAPFLCGPNHAA...QTLISEYRGNCPY. 308
PET6 DILGRNGVAWMKRFIDKDERYTQFLCGQNFDS...SLRVSEYRDNCSYY 297
IIb IsPETase ALIGKKGVAWMKRFMDNDTRYSTFACENPNSTRVSDFRTAN....CS.. 290
PbPL ALIGKYGVAWIKRFVDNDTRYSPFLCGAPHQADLRSSRLSEYRESCPY. 298
Mipa-P PIVSRAAVSWFKRFLDDDTRFAPFACDFGGAS......ISQFRSTCPV. 257
Kubu-P PTVSRAMVSWLKRFVSSDDRFTPFTCGFAGAA......VSAFRSTAC.. 252

B

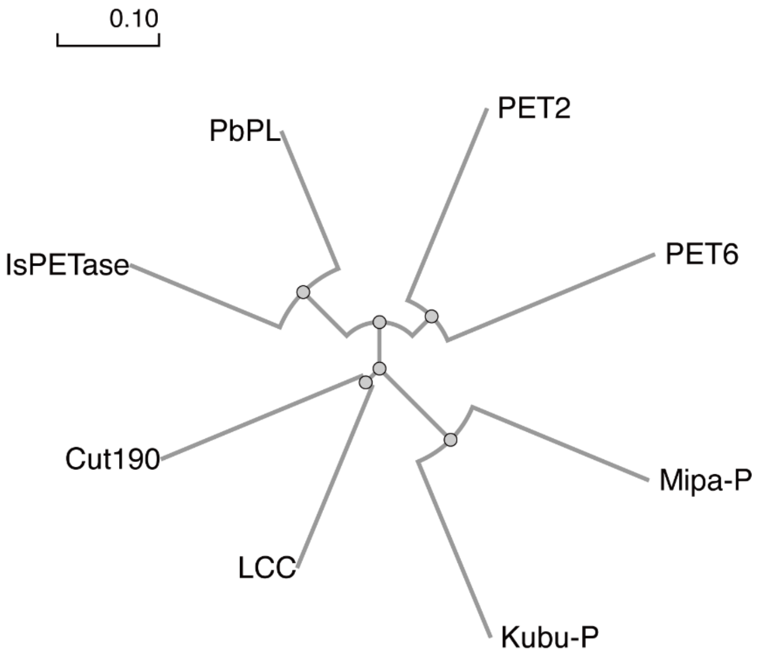

Figure S3. Multiple sequence alignment and phylogenic tree of Type I and II PET hydrolases, Mipa-P, and Kubu-P.

(**A**) Amino acid sequences of mature type I and II PET hydrolases (without signal peptides), Mipa-P, and Kubu-P (without amino-terminal methionine) were aligned using Clustal Omega. The resulting alignment was displayed using ESPript 3.0 (Robert and Gouet 2014). Residue numbering, including that of the signal peptide, is shown at the right of the sequence. Secondary structure elements are presented based on the crystal structure of LCC (PDB ID: 4EB0) (Sulaiman et al. 2014). The conserved residues are highlighted in boxes. Identical residues are marked with a red background, and highly conserved residues are shown in a red font. Black and white arrowheads indicate residues for catalytic triads and aromatic clumps, respectively. Closed and open circles indicate residues for subsites I and II, respectively. Green lines represent pairs of cysteine residues that form disulfide bonds. The green line represents a pair of cysteine residues forming a disulfide bond. The broken green line represents the second disulfide bond introduced into type II PET hydrolases. The protein accession numbers were as follows: LCC, HQ704839; Cut190, AB728484; PET2, ON416993; PET6, A0A1M5F0K3; *Is*PETase, WP_054022242; PbPL, WP_047194864. The sequences of Mipa-P and Kubu-P were obtained from Seo et al 2024.

(**B**) Phylogenic tree of Type I and II PET hydrolases, Mipa-P, and Kubu-P based on a Clustal Omega alignment.

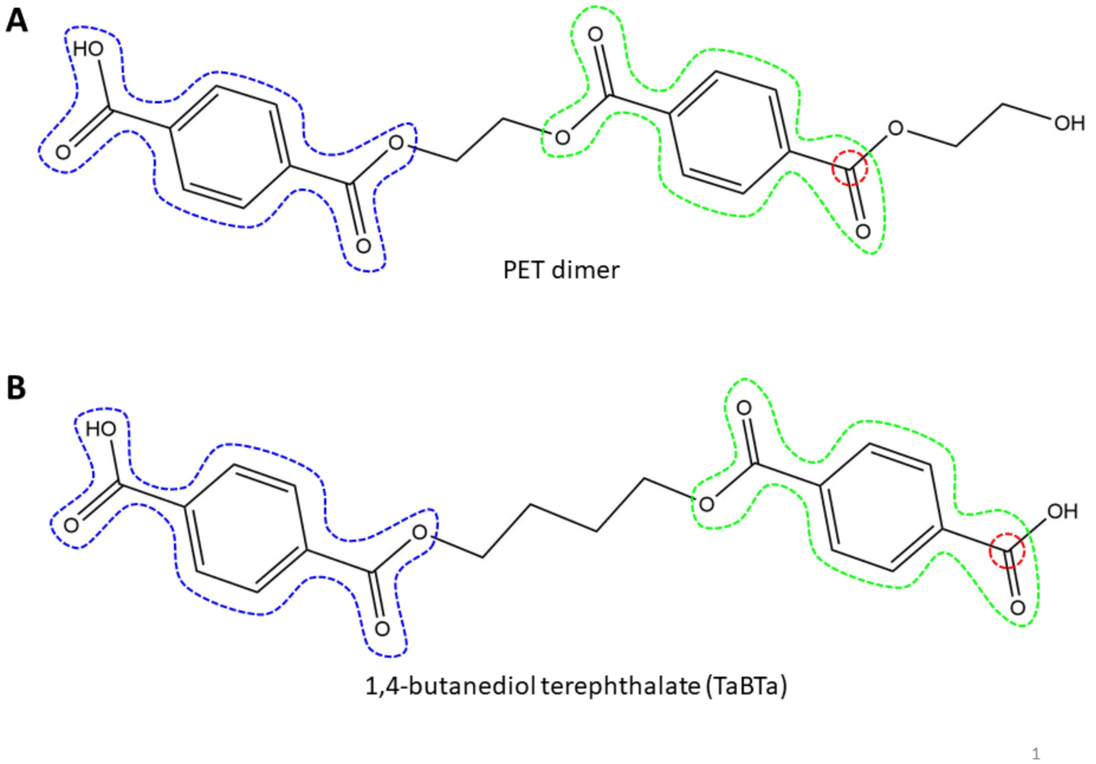

Figure S4. Chemical structures of ligands used for modeling with PET hydrolases. **A**. PET dimer. **B**. 1,4-butanediol diterephthalate (TaBTa), whose bound 3D structure on LCC$^{ICCG\text{-}S165A}$ is available as PDB ID: 8JMP. The red and green dotted curves show the corresponding atomic pairs between the two molecules, calculated by the topologically constrained disconnected maximum common substructure (TD-MCS) with θ=2. The red dotted circles are the scissile carbonyl group.

References

Austin HP, Allen MD, Donohoe BS, Rorrer NA, Kearns FL, Silveira RL, Pollard BC, Dominick G, Duman R, Omari KE, Mykhaylyk V, Wagner A, Michener WE, Amore A, Skaf MS, Crowley MF, Thorne AW, Johnson CW, Woodcock HL, McGeehan JE, Beckham GT (2018) Characterization and engineering of a plastic-degrading aromatic polyesterase. Proc Natl Acad Sci USA 115:E4350-E4357. doi: 10.1073/pnas.1718804115

Bell EL, Smithson R, Kilbride S, Foster J, Hardy FJ, Ramachandran S, Tedstone AS, High SJ, Garforth AA, Day PJR, Levy C, shaver MP, Green AP (2022) Directed evolution of an efficient and thermostable PET polymerase. Nat Catal 5:673-681. doi: 10.1038/s41929-022-00821-3

Bollinger A, Thies S, Knieps-Grünhagen E, Gertzen C, Kobus S, Höppner A, Ferrer M, Gohlke H, Smits SHJ, Jaeger K-E (2020) A novel polyester hydrolase from the marine bacterium *Pseudomonas aestusnigri*-structural and functional insights. Front Microbiol 11:114. doi: 10.3389/fmicb.2020.00114

Brinch-Pedersen W, Keller MB, Dorau R, Paul B, Jensen K, Borch K, Westh P (2024) Discovery and surface charge engineering of fungal cutinases for enhanced activity on poly(ethylene terephthalate). ACS Sustain Chem Eng 12:7329-7337. doi: 10.1021/acssuchemeng.4c00060

Case DA, Aktulga HM, Belfon K, Ben-Shalom IY, Brozell SR, Cerutti DS, Cheatham III TE, Cisneros GA, Cruzeiro VWD, Darden TA, Duke RE, Giambasu G, Gilson MK, Gohlke H, Goetz AW, Harris R, Izadi S, Izmailov SA, Jin C, Kasavajhala K, Kaymak MC, King E, Koval KP (2021) Amber 2021: Univ California, San Fr.

Chen CC, Han X, Li X, Jiang P, Niu D, Ma L, Liu W, Li S, Qu Y, Hu H, Min J, Y. Y, Zhang L, Zeng W, Huang JW, Dai L, Guo RY (2021) General features to enhance enzymatic activity of poly(ethylene terephthalate) hydrolysis. Nat Catal 4:425-430. doi: 10.1038/s41929-021-00616-y

Chen CC, Li X, Min J, Zeng Z, Ning Z, He H, Long X, Niu D, Peng R, Liu X, Yang Y. (2024) Complete decomposition of poly(ethylene terephthalate) by crude PET hydrolytic enzyme produced in *Pichia pastoris*. Chem Eng J 481: 148418. doi: 10.1016/j.cej.2023.148418

Chen X-Q, Guo Z-Y, Wang L, Yan Z-F, Jin C-X, Huang Q-S, Kong D-M, Rao D-M, Wu J (2022) Directional-path modification strategy enhances PET hydrolase catalysis of plastic degradation. J Hazard Mater 433:128816. doi: 10.1016/j.jhazmat.2022.128816

Chen Z, Duan R, Xiao Y, Wei Y, Zhang H, Sun X, Wang S, Cheng Y, Wang X, Tong S, Yao Y, Zhu C, Yang H, Wang Y, Wang Z (2022) Biodegradation of highly crystallized poly(ethylene terephthalate) through cell surface codisplay of bacterial PETase and hydrophobin. Nat Commun 13:7138. doi: 10.1038/s41467-022-34908-z

Cui Y, Chen Y, Liu X, Dong S, Tian Y, Qiao Y, Mitra R, Han J, Li C, Han X, Liu W, Chen Q, Wei W, Wang X, Du W, Tang S, Xiang H, Liu H, Liang Y, Houk KN, Wu B (2021) Computational redesign of PETase for plastic biodegradation under ambient condition by GRAPE strategy. ACS Catal 11:1340-1350. doi: 10.1021/acscatal.0c05126

Cui Y, Chen Y, Sun J, Zhu T, Pang H, Li C, Geng W-C, Wu B (2024) Computational redesign of a hydrolase for nearly complete PET depolymerization at industrially relevant high-solids loading. Nat Commun 15: 1417. doi: 10.1038/s41467-024-45662-9

Danso D, Schmeisser C, Chow J, Zimmermann W, Wei R, Leggewie C, Li X, Hazen T, Streit WR (2018) New insights into the function and global distribution of polyethylene terephthalate (PET)-degrading bacteria and enzymes in marine and terrestrial metagenomes. Appl Environ Microbiol 84: e02773-17. doi: 10.1128/AEM.02773-17

Eiamthong B, Meesawat P, Wongsatit T, Jitdee J, Sangsri R, Patchsung M, Aphicho K, Suraritdechachai S, Huguenin-Dezot N, Tang, S, Suginta W, Paosawatyanyong B, Babu MM, Chin JW, Palotiprapha D, Bhanthumnavin W, Uttamapinant C (2022) Discovery and genetic code expansion of a polyethylene terephthalate (PET) hydrolase from the human saliva metagenome for the degradation and bio-functionalization of PET. Angew Chem Int Ed 61:e202203061. doi: 10.1002/anie.202203061

Emori M, Numoto N, Senga A, Bekker G-J, Kamiya N, Kobayashi Y, Ito N, Kawai F, Oda M (2021) Structural basis of mutants of PET-degrading enzyme from *Saccharomonospora viridis* AHK190 with high activity and thermal stability. Proteins 89:502-511. doi: 10.1002/prot.26034

Erickson E, Gado JE, Avilán L, Aratti F, Brizendine R, Cox PA, Gill R, Graham R, Kim D-J, König G, Michener WE, Poudel S, Ramirez KJ, Shakespeare TJ, Zahn M, Boyd ES, Payne CM, Dubois JL, Pickford AR, Beckham GT, McGeehan JE (2022) Sourcing thermotolerant poly(ethylene terephthalate) hydrolase scaffolds from natural diversity. Nat Commun 13:7850. doi: 10.1038/s41467-022-35237-x

Fecker T, Galaz-Davison P, Engelberger F, Narui Y, Sotomayor M, Parra LP, Ramírez-Sarmíento CA (2018) Active site flexibility as a hallmark for efficient PET degradation by *I. sakaiensis* PETase. Biophys J 114:1302-1312. doi: 10.1016/j.bpj.2018.02.005

Furukawa M, Kawakami N, Tomizawa A, Miyamoto K (2019) Efficient degradation of poly(ethylene terephthalate) with *Thermobifida fusca* cutinase exhibiting improved catalytic activity generated using mutagenesis and additive-based approaches. Sci Rep 9:16038. doi: 10.1038/s41598-019-52379

Han X, Liu W, Huang J-W, Ma J, Zheng Y, Ko T-P, Xu L, Cheng Y-S, Chen C-C, Guo R-T (2018) Structural insight into catalytic mechanism of PET hydrolase. Nat Commun 8:2106. doi: 10.1038/s41467-017-02255-z

Herrero Acero E, Ribitsch D, Steinkellner G, Gruber K, Greimel K, Eiteljoerg I, Trotscha E, Wei R, Zimmermann W, Zinn M, Cavaco-Paulo A, Freddi G, Schwab H, Guebitz G (2011) Enzymatic surface hydrolysis of PET: Effect of structural diversity on kinetic properties of cutinases from *Thermobifida.* Macromolecules 44:4632-4640. doi: 10.1021/ma200949p

Hong H, Ki D, Seo H, Park J, Jang J, Kim K-J (2023) Discovery and rational engineering of PET hydrolase with both mesophilic and thermophilic PET hydrolase properties. Nat Commun 14:4556. doi: 10.1038/s41467-023-40233-w

Joho Y, Vongsouthi V, Spence MA, Ton J, Gomez C, Tan LL, Kaczmarski JA, Caputo AT, Royan S, Jackson

CJ, Ardevol A (2023) Ancestral sequence reconstruction identifies structural changes underlying the evolution of *Ideonella sakaiensis* PETase and variants with improved stability and activity. Biochemistry 62:437-450. doi: 10.1021/acs.biochem.2c00323

Joo S, Cho IJ, Seo H, Son HF, Sagong HY, Shin TJ, Choi SY, Lee SY, Kim KJ (2018) Structural insight into molecular mechanism of poly(ethylene terephthalate) degradation. Nat Commun 9:382. doi: 10.1038/s41467-018-02881-1

Kawabata T (2011) Build-up algorithm for atomic correspondence between chemical structures. J Chem Inf Model 51:1775-1787. doi:10.1021/ci2001023

Kawabata T, Nakamura H (2014) 3D flexible alignment using 2D maximum common substructure: dependence of prediction accuracy on target-reference chemical similarity. J Chem Inf Model 54:1850-1863. doi:10.1021/ci500006d

Kawabata T, Nishikawa K (2000) Protein structure comparison using the Markov transition model of evolution. Proteins 41:108-122. doi: 10.1002/1097-0134(20001001)41:1<108::AID-PROT130>3.0.CO;2-S

Kawai F, Furushima Y, Mochizuki N, Muraki N, Yamashita M, Iida A, Mamoto R, Tosha T, Iizuka R, Kitajima S (2022) Efficient depolymerization of polyethylene terephthalate (PET) and polyethylene furanoate by engineered PET hydrolase Cut190. AMB Express 12:134. doi: 10.1186/s13568-022-01474-y

Kawai F, Oda M, Tamashiro T, Waku T, Tanaka N, Yamamoto M, Mizushima H, Miyakawa T, Tanokura M (2014) A novel Ca^{2+}-activated, thermostabilized polyesterase capable of hydrolyzing polyethylene terephthalate from *Saccharomonospora viridis* AHK190. Appl Microbiol Biotechnol 98:10053-10064. doi: 10.1007/s00253-014-5860-y

Lee SH, Seo H, Hong H, Park J, Ki D, Kim M, Kim HJ, Kim KJ (2023) Three-directional engineering of *Is*PETase with enhanced protein yield, activity, and durability. J Hazard Mater 459:132297. doi: 10.1016/j.jhazmat.2023.132297

Li X, Shi B, Huang JW, Zeng Z, Yang Y, Zhang L, Min J, Chen CC, Guo RT (2023) Functional tailoring of a PET hydrolytic enzyme expressed in *Pichia pastoris*. Bioresour Bioprocess 10:26. doi: 10.1186/s40643-023-00648-1

Liu B, He L, Wang L, Li T, Li C, Liu H, Luo Y, Bao R (2018) Protein crystallography and site-direct mutagenesis analysis of the poly(ethylene terephthalate) hydrolase PETase from *Ideonella sakaiensis.* Chembiochem 19: 1471-1475. doi: 10.1002/cbic.201800097

Liu C, Shi C, Zhu S, Wei R, Yin CC (2019) Structural and functional characterization of polyethylene terephthalate hydrolase from *Ideonella sakaiensis.* Biochem Biophys Res Commun 508:289-294. doi: 10.1016/j.bbrc.2018.11.148

Lu H, Diaz DJ, Czarnecki NJ, Zhu C, Kim W, Schroff R, Acosta DJ, Alexander BR, Cole HO, Zhang Y, Lynd NA, Ellington AD, Alper HS (2022) Machine leaning-aided engineering of hydrolases for PET

depolymerization. Nature 604:662-667. doi: 10.1038/s41586-022-04599-z

Meilleur C, HupéJF, Juteau P, Shareck F (2009) Isolation and characterization of a new alkali-thermostable lipase cloned from a metagenomic library. J Ind Microbiol Biotechnol 36:853-861. doi: 10.1007/s10295-009-0562-7

Miyakawa T, Mizushima H, Ohtsuka J, Oda M, Kawai F, Tanokura M (2014) Structural basis for the Ca^{2+}-enhanced thermostability and activity of PET-degrading cutinase-like enzyme from *Saccharomonospora viridis* AHK190. Appl Microbiol Biotechnol 99:4297-4307. doi: 10.1007/s00253-014-6272-8

Nakamura A, Kobayashi N, Koga N, Iino R (2021) Positive charge introduction on the surface of thermostabilized PET hydrolase facilitates PET binding and degradation. ACS Catal 11:8550-8564. doi: 10.1021/acscatal.1c01204

Numoto N, Kamiya N, Bekker G-J, Yamagami Y, Inaba S, Ishii K, Uchiyama S, Kawai F, Ito N, Oda M (2018) Structural dynamics of the PET-degrading cutinase-like enzyme from *Saccharomonospora viridis* AHK190 in substrate-bound states elucidates the Ca^{2+}-driven catalytic cycle. Biochemistry 57:5289-5300. doi: 10.1021/acs.biochem.8b00624

Numoto N, Kamiya N, Oda M (2023) Improvement of thermostability and activity of PET-degrading enzyme Cut190 towards a detailed understanding and application of the enzymatic reaction mechanism. bioRxiv. doi: 10.1101/2023.02.26.529345

Oda M, Yamagami Y, Inaba S, Oida I, Yamamoto M, Kitajima S, Kawai F (2018) Enzymatic hydrolysis of PET: Functional roles of three Ca^{2+} ions bound to a cutinase-like enzyme, Cut190*, and its engineering for improved activity. Appl Microbiol Biotechnol 102:10067-10077. doi: 10.1007/s00253-018-9374-x

Palm GJ, Reisky L, Böttcher D, Műller H, Michels EAP, Walczak MC, Berndt L, Weiss MS, Bornscheuer UT, Weber G (2019) Structure of the plastic-degrading *Ideonella sakaiensis* MHETase bound to a substrate. Nat Commun 10:1717. doi: 10.1038/s41467-019-09326-3

Pettersen EF, Goddard TD, Huang CC, Couch GS, Greenblatt DM, Meng EC, Ferrin TE (2004) UCSF Chimera—a visualization system for exploratory research and analysis. J Comput Chem 25:1605-1612. doi: 10.1002/jcc.20084

Pfaff L, Gao J, Li Z, Jäcjerubg A, Weber G, Mican, J, Chen Y, Dong W, Han X, Feiler CG, Ao Y-F, Badenhorst CPS, Bednar D, Palm G, Lammers M, Damborsky J, Strodel B, Liu W, Bornscheuer UT, Wei, R (2022) Multiple substrate binding mode-guided engineering of a thermophilic PET hydrolase. ACS Catal 12:9790-9800. doi: 10.1021/acscatal.2c02275

Ribitsch D, Hromic A, Zitzenbacher S, Zartl B, Gamerith C, Pellis A, Jungbauer A, Lyskowski A, Steinkellner G, Gruber K, Tscheliessnig R, Acero EH, Guebitz GM (2017) Small cause, large effect: structural characterization of cutinases from *Thermobifida cellulosilytica.* Biotechnol Bioeng 114:2481-2488. doi: 10.1002/bit.26372

Robert X, Gouet P (2014) Deciphering key features in protein structures with the new ENDscript server.

Nucleic Acids Res 42:W320-324. doi: 10.1093/nar/gku316

Roth C, Wei R, Oeser T, Then J, Föller C, Zimmermann W, Sträter N (2014) Structural and functional studies on a thermostable polyethylene terephthalate degrading hydrolase from *Thermobifida fusca*. Appl Microbiol Biotechnol 98:7815-7823. doi: 10.1007/s00253-014-5672-0

Senga A, Numoto N, Yamashita M, Iida A, Ito N, Kawai F, Oda M (2021) Multiple structural states of Ca^{2+}-regulated PET hydrolase, Cut190, and its correlation with activity and stability. J Biochem 169:207-214. doi: 10.1093/jb/mvaa102

Seo H, Hong H, Part J, Lee SH; Ki D, Ryu A, Sagong HY, Kim KJ (2024) Landscape profiling of PET depolymerases using a natural sequence cluster framework. bioRxiv doi: 10.1101/2024.04.01.587509

Shi L, Liu P, Tan Z, Zhao W, Gao J, Gu Q, Ma H, Liu H, Zhu L (2023) Complete depolymerization of PET wastes by an evolved PET hydrolase from directed evolution. Angew Chem Int Ed 62:e202218390. doi: 10.1002/anie.202218390

Son HF, Cho IJ, Joo S, Seo H, Sagong H-Y, Choi SY, Lee SY, Kim K-J (2019) Rational protein engineering of thermos-stable PETase from *Ideonella sakaiensis* for highly efficient PET degradation. ACS Catal 9:3519-3526. doi: 10.1021/acscatal.9b00568

Sonnendecker C, Oeser, J, Richter PK, Hille P, Zhao Z, Fischer C, Lippold H, Blázquez-Sánchez P, Engelberger, F, Ramírez-Sarmiento CA, Oeser T, Lihanova Y, Frank R, Jahnke H-G, Billig S, Abel B, Sträter N, Matysik J, Zimmerman W (2022) Low carbon Footprint recycling of post-consumer PET plastic with a metagenomic polyester hydrolase. ChemSusChem 15:e202101062. doi: 10.1002/cssc.202101062

Sulaiman S, Yamato S, Kanaya E, Kim J-J, Koga Y, Takano K, Kanaya S (2012) Isolation of a novel cutinase homolog with polyethylene terephthalate-degrading activity form leaf-branch compost by using metagenomic approach. Appl Environ Microbiol 78:1556-1562. doi: 10.1128/AEM.06725-11

Sulaiman S, You D-J, Kanaya E, Koga Y, Kanaya S (2014) Crystal structure and thermodynamic and kinetic stability of metagenome-derived LC-cutinase. Biochemistry 53:1858-1869. doi: 10.1021/bi401561p

Then J, Wei R, Oeser T, Gerdts A, Shmidt J, Barth M, Zimmermann W (2016) A disulfide bridge in the calcium binding site of a polyester hydrolase increases its thermal stability and activity against polyethylene terephthalate. FEBS Open Bio 6:425-432. doi: 10.1002/2211-5463.1205

Tournier V, Duquesne S, Guillamot F, Cramail H, Taton D, Marty A, André I (2023) Enzymes' Power for Plastics Degradation. Chem Rev 123:5612-5701. doi: 10.1021/acs.chemrev.2c00644

Tournier V, Topham CM, Gilles A, David B, Folgoas C, Moya-Leclair E, Kamionka E, Desrousseaux ML, Texier H, Gavalda S, Cot M, Guémard E, Dalibey M, Nomme J, Cioci G, Barbe S, Chateau M, André I, Duquesne S, Marty A (2020). An engineered PET depolymerase to break down and recycle plastic bottles. Nature 580:216-219. doi: 10.1038/s41586-020-2149-4

Wei R, Oeser T, Schmidt J, Meier R, Barth M, Then J, Zimmermann W (2016) Engineered bacterial

polyester hydrolases efficiently degrade polyethylene terephthalate due to relieved product inhibition. Biotechnol Bioeng 113:1658-1664. doi: 10.1002/bit.25941

Xi X, Ni K, Hao H, Shang Y, Zhao B, Qian Z (2021) Secretary expression in *Bacillus subtilis* and biochemical characterization of a highly thermostable polyethylene terephthalate hydrolase from bacterium HR29. Enzyme Microb Technol 143:109715. doi: 10.1016/j.enzmictec.2020.109715

Yang Y, Cheng S, Zheng Y, Xue T, Huang J-W, Zhang L, Yang Y, Guo R-T, Chen C-C (2024) Remodeling the polymer-binding cavity to improve the efficacy of PBAT-degrading enzyme. J Hazard Mater 464: 132965. doi: 10.1016/j.hazmat.2023.132965

Yang Y, Min J, Xue T, Jiang P, Liu X, Peng R, Huang JW, Qu Y, Li X, Ma N, Tsai FC, Dai L, Zhang Q, Liu Y, Chen CC, Guo RT (2023) Complete bio-degradation of poly(butylene adipate-co-terephthalate) via engineered cutinases. Nat Commun 14:1645. doi: 10.1038/s41467-023-37374-3

Yin Q, Zhang J, Ma S, Gu T, Wang M, You S, Ye S, Su R, Wang Y, Qi W (2024) Efficient polyethylene terephthalate biodegradation by an engineered *Ideonella sakaiensis* PETase with a fixed substrate-binding W156 residue. Green Chem 26: 2560-2570. doi: 10.1039/D3GC03663D

Yoshida S, Hiraga K, Takehana T, Taniguchi I, Yamaji H, Maeda Y, Toyohara K, Miyamoto K, Kimura Y, Oda K (2016) A bacterium that degrades and assimilates poly(ethylene terephthalate). Science 351:1196-1199. doi: 10.1126/science.aad6359

Zeng W, Li X, Yang Y, Min J, Huang J-W, Liu W, Niu D, Yang X, Han X, Zhang L, Dai L, Chen C-C, Guo R-T (2022) Substrate-binding mode of a thermophilic PET hydrolase and engineering the enzyme to enhance the hydrolytic efficiency. ACS Catal 12:3033-3040. doi: 10.1021/acscatal.1c05800

Zhang Z, Huang S, Cai D, Shao C, Zhang C, Zhou J, Cui Z, He T, Chen C, Chen B, Tan T (2022) Depolymerization of post-consumer PET bottles with engineered cutinase 1 from *Thermobifida cellusilytica.* Green Chem 24:5998-6007. doi: 10.1039/d2gc01834a

Zheng Y, Li Q, Liu P, Yuan Y, Dian L, Wang Q, Lian Q, Su T, Qi Q (2024) Dynamic docking-assisted engineering of hydrolases for efficient PET depolymerization. ACS Catal 14:3627-3639. doi: 10.1021/acscatal.4c00400
